# Dynamics of gene expression during development and expansion of vegetative stem internodes of bioenergy sorghum

**DOI:** 10.1186/s13068-017-0848-3

**Published:** 2017-06-21

**Authors:** Tesfamichael H. Kebrom, Brian McKinley, John E. Mullet

**Affiliations:** Department of Biochemistry and Biophysics, Texas A&M University, College Station, TX 77843 USA

**Keywords:** Bioenergy, Sorghum, Internode, Transcriptome, Cell cycle, Hormone

## Abstract

**Background:**

Bioenergy sorghum accumulates 75% of shoot biomass in stem internodes. Grass stem internodes are formed during vegetative growth and elongate in response to developmental and environmental signals. To identify genes and molecular mechanisms that modulate the extent of internode growth, we conducted microscopic and transcriptomic analyses of four successive sub-apical vegetative internodes representing different stages of internode development of the bioenergy sorghum genotype R.07020.

**Results:**

Stem internodes of sorghum genotype R.07020 are formed during the vegetative phase and their length is enhanced by environmental signals such as shade and floral induction in short days. During vegetative growth, the first visible and youngest sub-apical internode was ~0.7 cm in length, whereas the fourth fully expanded internode was ~5 cm in length. Microscopic analyses revealed that all internode tissue types including pith parenchyma and vascular bundles are present in the four successive internodes. Growth in the first two sub-apical internodes occurred primarily through an increase in cell number consistent with expression of genes involved in the cell cycle and DNA replication. Growth of the 3rd internode was associated with an increase in cell length and growth cessation in the 4th internode was associated with up-regulation of genes involved in secondary cell wall deposition. The expression of genes involved in hormone metabolism and signaling indicates that GA, BR, and CK activity decreased while ethylene, ABA, and JA increased in the 3rd/4th internodes. While the level of auxin appears to be increasing as indicated by the up-regulation of ARFs, down-regulation of TIR during development indicates that auxin signaling is also modified. The expression patterns of transcription factors are closely associated with their role during the development of the vegetative internodes.

**Conclusions:**

Microscopic and transcriptome analyses of four successive sub-apical internodes characterized the developmental progression of vegetative stem internodes from initiation through full elongation in the sorghum genotype R.07020. Transcriptome profiling indicates that dynamic variation in the levels and action of GA, CK, IAA, BR, ethylene, ABA, and JA modulate gene expression and growth during internode growth and development. This study provides detailed microscopic and transcriptomic data useful for identifying genes and molecular pathways regulating internode elongation in response to various developmental and environmental signals.

**Electronic supplementary material:**

The online version of this article (doi:10.1186/s13068-017-0848-3) contains supplementary material, which is available to authorized users.

## Background

The C4 grasses are the world’s most important source of sucrose [1], and bioethanol, and have great promise as bioenergy crops due to their high biomass yield (~20 to 50 dry Mg/ha) [2–6]. High biomass C4 grasses such as sorghum, sugarcane, Napier grass, and Miscanthus accumulate most of their harvestable biomass in stems [1, 2, 6–8]. The stems of these C4 grasses are composed of a series of nodes and internodes. A node and an internode are formed as part of a phytomer, which includes a leaf and an axillary bud initiated from cells in the peripheral and rib zones of the shoot apical meristem (SAM). Once initiated, internodes elongate by increasing cell numbers through cell division and by cell elongation. In plants with similar flowering times, stem length is highly correlated with the average length of internodes. A longer internode of similar diameter has a higher biomass and can accumulate more sugar. Therefore, increasing the length of internodes can potentially increase sink strength and the biomass yield of C4 grass bioenergy crops.

Internode elongation is regulated by genetic, developmental, and environmental factors. Many of the genes controlling internode elongation were identified by studying plant height mutants in diverse species. The genes identified encode proteins involved in hormone metabolism, transport, and signaling among other functions. Plant hormones such as auxin, strigolactones, GA, and BR promote, whereas ABA and JA inhibit stem elongation. In sorghum, mutation of *Dw3*, an ABCB1 auxin efflux carrier expressed in stem nodes, and *Dw1*, a plasma membrane protein, reduce internode length [9, 10]. Ethylene either promotes or inhibits internode elongation depending on the genotype, environmental conditions, and developmental status of internodes and the whole plant. For example, in rice, submergence either induces or inhibits internode elongation depending on genotype, responses mediated in part through an increase in the level of ethylene [11–14]. Complex cross-talk between plant hormones regulates stem growth and development. For example, auxin promotes internode elongation in part by controlling the level of GA and ethylene regulates the level of GA or ABA to induce or repress internode elongation, respectively. Since more than one plant hormone regulates internode elongation, their effect depends on the relative level of each hormone, plant tissue, stage of development, and environmental conditions.

Basal internodes formed during the juvenile phase in grasses do not elongate. In maize, rice, and Arabidopsis, internode elongation increases after the transition of the shoot apical meristem (SAM) from vegetative to flowering stage. The inhibition of internode elongation during the vegetative phase in these species has been linked to expression of *GA2oxidases* (*GA2oxs*) that inactivate GA at the base of the SAM [15–17]. In addition to promoting internode elongation, GA is also florigenic; therefore, GA2oxs protect the SAM from GA that induces transition to reproductive stage. In early flowering grain sorghum, minimal internode elongation occurs until floral initiation; however, internode elongation is observed during the vegetative phase in later flowering genotypes [18–20]. Energy sorghum hybrids that undergo floral initiation 150 days after planting are tall with long internodes indicating that non-florigenic signals can induce internode elongation in these vegetative plants. Shade signals, perceived mainly by the red and far red (FR) absorbing photoreceptor phytochrome B (PhyB), are one of the major environmental factors that modulate internode elongation. Developmental and environmental signals promote internode elongation in part by regulating hormone metabolism and signaling.

The focus of previous studies of internode gene expression was on cell wall and lignin biosynthesis. Ehlting et al. investigated gene expression during different stages of vascular and interfascicular differentiation in stems of Arabidopsis and identified genes associated with secondary wall formation and lignification [21]. Furthermore, the gene regulatory network for secondary cell wall biosynthesis in Arabidopsis was elucidated using mutants, developmental time course studies, and other co-expression studies [22, 23]. In maize, genes involved in cell wall biosynthesis were identified by profiling gene expression of elongating and fully elongated stem internodes [24]. The study of different regions of elongating rice and Setaria stem internodes helped identify a network of genes involved in secondary cell wall biosynthesis [25, 26]. In addition, a prior study examined changes in gene expression that occur in sweet sorghum stems from floral initiation through post-grain maturity [27]. However, gene expression during vegetative growth and development of C4 grass internodes has not been investigated in detail. In particular, in bioenergy C4 grasses such as energy sorghum where stem biomass is the major yield component, detailed information on vegetative stem growth, development, and gene expression may be useful for improving biomass yield and composition [2, 3, 28].

Sorghum has emerged as an excellent genetic system for the design of C4 bioenergy grasses and as a source of drought resilient commercial bioenergy hybrids [2, 3, 6]. Sorghum is a diploid inbreeding species with a diverse germplasm enabling genetic analysis, gene discovery, and hybrid breeding [2, 3]. Numerous sorghum genome sequences are now available [29–31], and sorghum transformation efficiency has recently improved significantly [32].

Detailed characterization of the development of the vegetative internodes from initiation, elongation, and differentiation could reveal molecular mechanisms that regulate the growth of the vegetative internodes. Such studies will provide the basis for further analysis of molecular mechanisms and genes modulated by developmental and environmental signals that modulate the extent of internode elongation. Therefore, in this paper, we report microscopic and transcriptome analyses of four successive sub-apical internodes of the bioenergy sorghum genotype R.07020 that represent different stages of internode development. The results show that all tissue types of an internode including the vascular bundles are present in the youngest sub-apical internode and undergo further development in the successive internodes. Furthermore, cell division enhances growth in the first two sub-apical internodes; cell length increases in the third internode, and lignin accumulates in the fourth full-length internode. Physiological and molecular mechanisms that regulate the elongation of vegetative internodes in the C4 grass sorghum are discussed.

## Methods

### Plant material and growing conditions

**Fig. 1:**
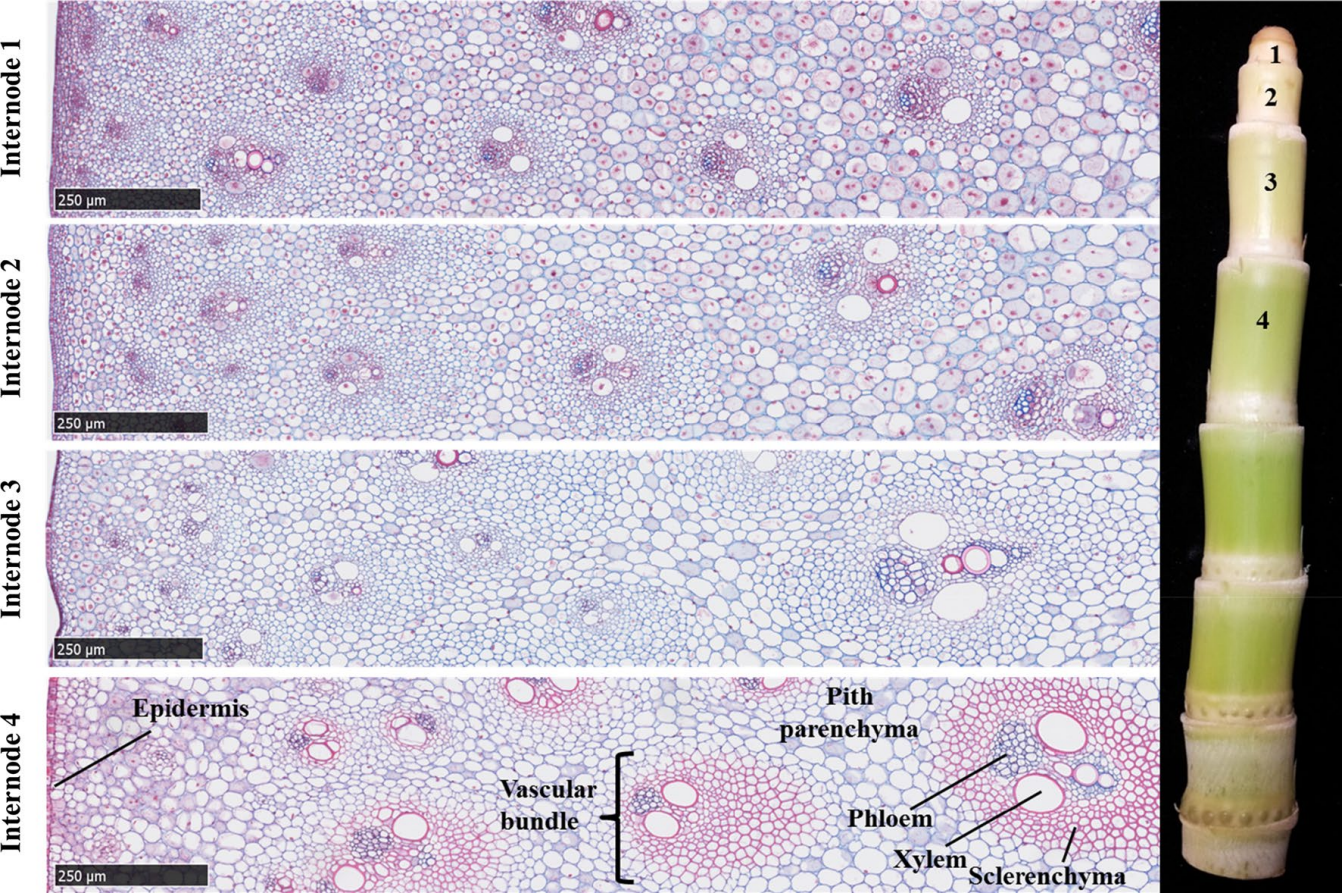
Microscopic cross sections of internodes of bioenergy sorghum inbred R.07020 at 60 DAP (days after planting). Internode1 (Int1) is the youngest sub-apical internode and internode 4 (Int4) is the oldest fully expanded internode. Stem sections were stained with *safranin* and *alcian blue*. Samples were from the middle section of each internode

For microscopic and RNA-seq analysis of successive sub-apical internodes of sorghum stems, the photoperiod-sensitive bioenergy genotype R.07020 was grown in a growth chamber for 60 days under long-day conditions (14-h photoperiod) to prevent floral induction. The growth chamber’s temperature was 31/22 °C day/night and at constant 50% relative humidity. The light in the growth chamber was supplied with fluorescent lamps at a light intensity of ~350 µMol/m^2^/s of photosynthetically active radiation (PAR). At 60 DAP internodes were harvested from three different plants for analysis. Leaves and leaf sheaths were removed prior to sampling stem tissue for microscopic and RNA-seq analyses. At this stage of development, the upper portion of the stem consists of a dome of tissue surrounding the shoot apical meristem, followed by the first visible internode (Fig. 1 right, labeled 1). The next three internodes were successively larger in diameter and length (Fig. 1 Int1–4).

### Microscopic studies of internode development

For microscopic studies of internode development, cross sections and longitudinal sections were obtained from the middle section of the first four sub-apical internodes and fixed in FAA overnight. The tissues were transferred into 70% ethanol, embedded in paraffin, sectioned to 10 µm, and stained with alcian blue and safranin. Digital images (20×) of the slides were obtained using a Nanozoomer HT slide scanner system (Hamamatsu Photonics, Japan). Tissue embedding, sectioning, and digital imaging of the slides were performed at the Veterinary School histology and gastrointestinal laboratories at Texas A&M University. The digital images were viewed using NDP.view2 software (Hamamatsu Photonics, Japan).

### RNA-seq library sequencing, mapping, and statistical analysis

For RNA-seq library preparation, the first visible sub-apical internode below the apical dome and three successive internodes below the sub-apical internode (Fig. 1 right) were extracted from three plants and frozen in liquid nitrogen. RNA was extracted using TRIzol according to the manufacturer’s protocol (Invitrogen). About three µg of total RNA was used for RNA-seq library preparation using TruSeq™ RNA Sample Prep Kit v2 (Illumina, Inc.). RNA-seq library quality was assessed using a Bioanalyzer and sequenced on an Illumina HiSeq2500 at the Texas A&M University Genomics and Bioinformatics Service Center. The sequence reads were aligned with the sorghum transcript sequence *Sorghum bicolor* v3.1 (DOE-JGI, http://phytozome.jgi.doe.gov/) using the CLC Genomics Workbench (CLC bio, Denmark). The *Sbicolor*-*v3.1* contains 47,205 protein-coding transcripts. The RNA-seq data were analyzed using the EdgeR software in the CLC genomics workbench (CLC bio, Denmark), which generated expression values, RPKM (reads per kilobase of transcript per million mapped reads), fold change, *p* value, and FDR (false discovery rate corrected *p* value). The FDR in CLC bio is calculated using the Benjamini and Hochberg method [33]. The quality of the RNA-seq data was assessed using principal component analysis (PCA) in the CLC genomics workbench. The original expression values were used for PCA. A list of transcripts with mean RPKM ≥1 in each internode was classified as expressed genes [34, 35], and displayed in a Venn diagram using Venny 2.0 [36]. The RPKM values of transcripts in each internodes differentially expressed at twofold or higher between any pair of internodes with mean RPKM ≥2 and FDR <0.001 were clustered in MultiExperiment Viewer (MeV) using K-means clustering [37]. The RPKM values for each gene in each internode were normalized in MeV prior to K-means clustering. The figures of merit method (FOM) in MeV were used to determine the number of clusters. The Sorghum *bicolor v3.1* transcripts were annotated using Mercator by a Blast search against the Arabidopsis proteome to create a mapping file to initially categorize differentially expressed genes by the MapMan software [38, 39]. The list of transcripts in each cluster was annotated and classified using the MapMan software [38].

### Validation of RNA-seq data using quantitative real-time PCR (qRT-PCR)

To validate the RNA-seq data by qPCR, RNA was extracted from the same tissues used for RNA-seq using TRIzol according to the manufacturer’s protocol (Invitrogen). cDNAs were prepared from 1.5 µg of total RNA from three independent biological replicates for each sample. The RNAs were treated with DNase I (Invitrogen). Half of the DNase I-treated RNA was used to synthesize cDNA (+RT) using SuperScript III (Invitrogen) in a 20 µl reaction mix following the manufacturer’s protocol while the remaining half was used as a control (−RT control). The cDNA was suspended in 320 µl of water and 2 µl was used in qRT-PCR reactions. In addition to the cDNA template, the qRT-PCR reaction included 2 µl of 250 ng primer pairs (Additional file 1) and 6 µl KiCqStart^®^ SYBR Green qRT-PCR Ready Mix (Sigma-Aldrich). The 10 µl qPCR reaction was run in duplicate on an ABI 7900HT, with the corresponding −RT control. The average target cT (threshold cycle) values were normalized to 18s rRNA cT values. The fold change between two samples was calculated using the slightly modified 2^−(ΔΔCt)^ method as described in [40].

## Results

### Growth and development of vegetative internodes of bioenergy sorghum genotype R.07020

Bioenergy sorghum inbred R.07020 internodes are formed during the vegetative phase (Additional file 2). In the field, R.07020 plants that develop when day lengths exceed 12.4 h remain vegetative for approximately 150 days until day lengths are less than 12.4 h in mid-September. At 60 DAP, R.07020 plants grown at low density in growth chambers under 14-h day lengths had developed 14 fully expanded leaves. Continued growth under these conditions for an additional 18 days resulted in plants with elongated upper internodes (Additional file 2). When 60 DAP plants were grown for an additional 18 days in short days (10 h) to induce floral initiation or were exposed to increased shading by increasing plant density, internode elongation increased, especially in response to shading (Additional file 2). These results indicate that R.07020 elongates upper stem internodes during the vegetative phase and that shading can modify the extent of internode elongation during vegetative growth.

**Fig. 2:**
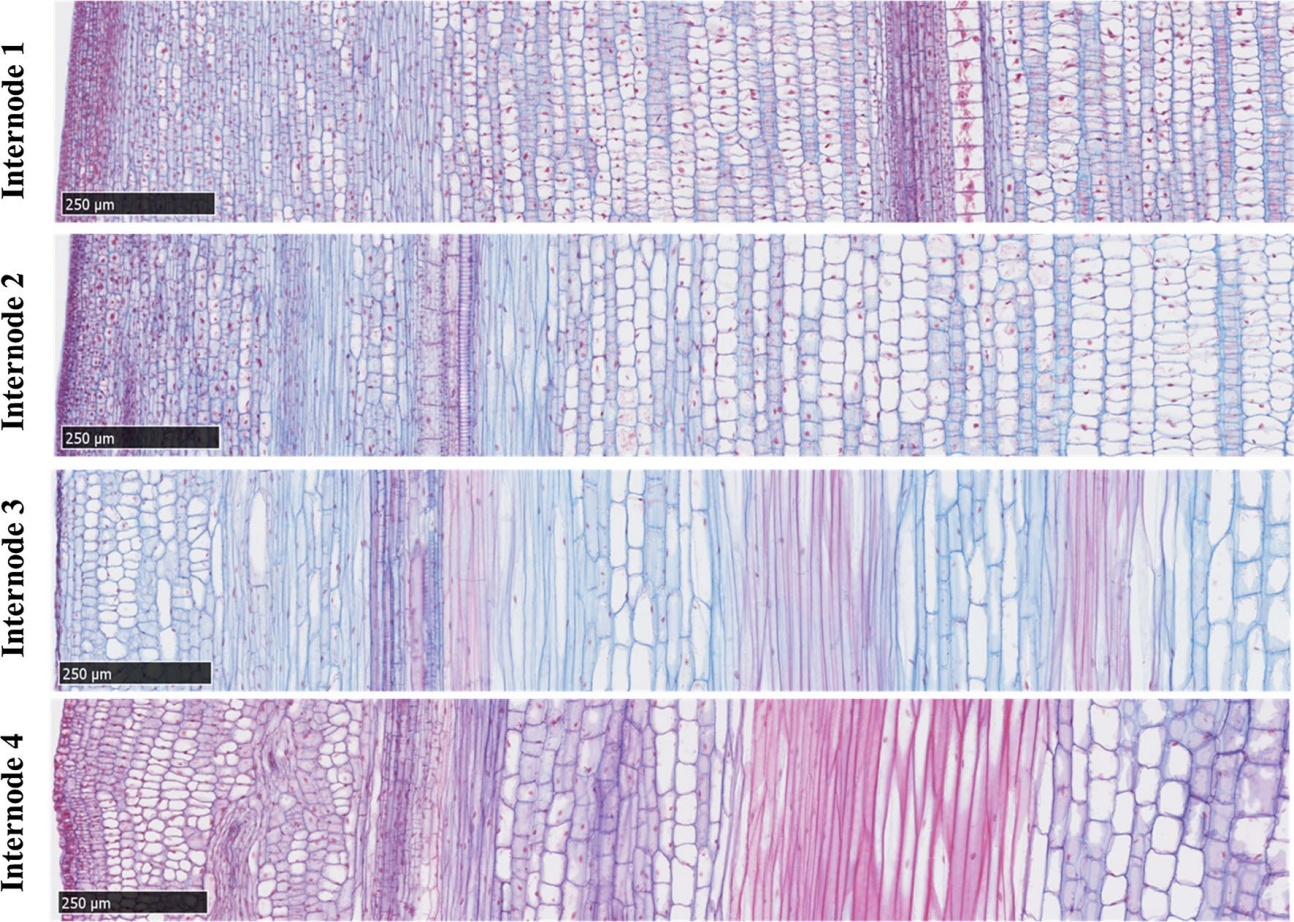
Microscopic longitudinal sections of internodes of bioenergy sorghum inbred R.07020 at 60 DAP (days after planting). Internode1 (Int1) is the youngest sub-apical internode, and internode 4 (Int4) is the oldest fully expanded internode. Stem sections were stained with *safranin* and *alcian blue*. Samples were from the middle section of each internode

A baseline of data on internode development and gene expression was collected from R.07020 plants grown in long days at low density for 60 days. In these plants, the average lengths of the four successive sub-apical internodes (Int1–4) below the shoot apex were 0.7, 2, 4.5, and 5, respectively. Cross sections of the four internodes taken approximately at the mid-point between nodes showed the presence of vascular bundles (VBs), pith parenchyma (PP), and epidermal cells (EC) at all stages of development (Fig. 1 left, labeled). Based on cell diameter and lignification, tissues in Int4 are more fully differentiated than the younger upper internodes. Xylem and PP cells were largest in diameter and cells surrounding VBs and cells located close to the epidermis were smaller in diameter. Lignification, revealed by staining cross sections with safranin, was highest in Int4 in sclerenchyma cells surrounding VBs (Fig. 1 red-stained cells). Phloem cells of the VB and PP cells were not stained with safranin indicating lower levels of lignin in their cell walls. Longitudinal sections of internodes revealed a large increase in the length of cells between Int2 and Int3 (Fig. 2). Cells associated with VBs were shorter in Int1, and of increasing length in Int2, 3, and 4.

### Transcriptome analysis of stem internode development

**Fig. 3:**
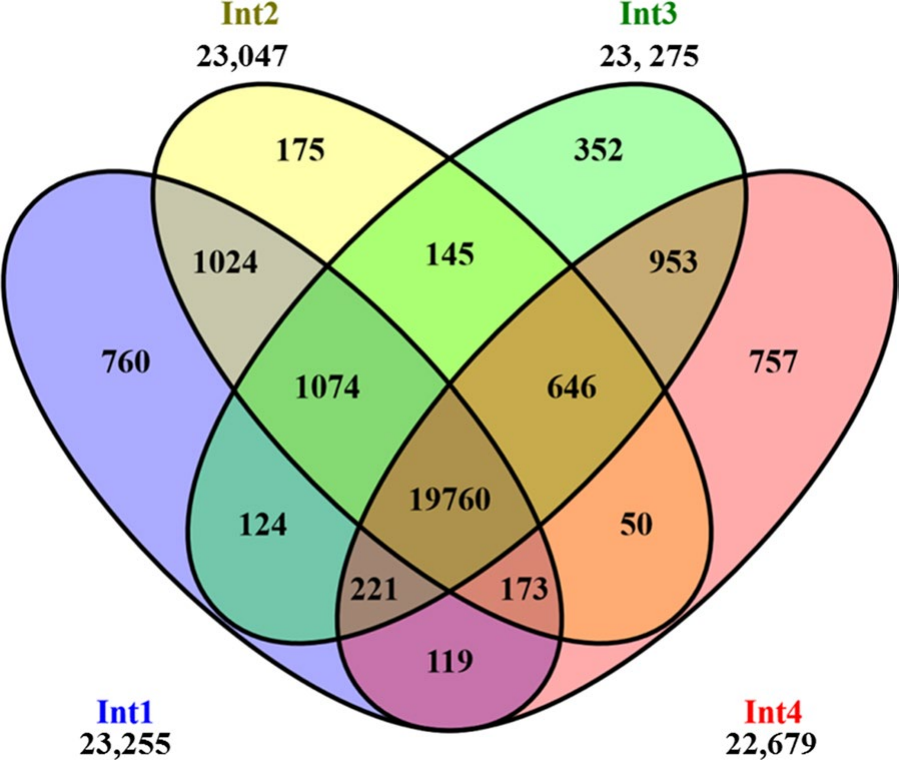
Venn diagram displaying genes expressed in four successive sub-apical internodes of bioenergy sorghum inbred R.07020. Int1 is the youngest sub-apical and Int4 is the oldest internode

The four successive internodes below the shoot apex were excised at 60 days after planting (DAP) from three plants (independent biological replicates) for analysis of transcriptome profiles using RNA-seq technology. A total of 153.2 million sequences were generated from the four successive sub-apical internodes. The total single RNA-seq reads generated from three independent biological replicates from Int1, Int2, Int3, and Int4 ranged from 34.6 to 41.5 million per sample (Additional file 3). The sequences were aligned to the *Sbicolor*-*v3.1* reference sequence (Phytozome) comprising 47,205 protein-coding transcripts using the CLC genomics workbench (CLC Inc., Aarhus, Denmark). On average about 90.2% of the reads were uniquely mapped; 3.3% were mapped non-specifically, and 6.5% of the reads were not mapped to the reference sequence (Additional file 3). The quality of the RNA-seq data was analyzed using principal component analysis (Additional file 4). The biological replicates within a sample clustered together and diverged from clusters from successive internode samples. The number of transcripts expressed in each internode was determined using a mean RPKM ≥1.00 cut-off. The number of transcripts expressed in Int1, Int2, Int3, and Int4 was 23,255, 23,047, 23,275, and 22,679, respectively (Fig. 3) and 19,760 transcripts were expressed in common between the four internodes. The number of transcripts expressed with an RPKM ≥1.00 in only one of the four internodes, and thus expressed exclusively in Int1, Int2, Int3, or Int4 were 760, 175, 352, and 757, respectively (Fig. 3). Most of these transcripts were expressed at low level with RPKM less than two (Additional file 5).

Differentially expressed genes were analyzed using EdgeR in the CLC genomics workbench (CLC Inc., Aarhus, Denmark). About 8361 transcripts were differentially expressed at twofold or higher (FDR <0.001 and average RPKM ≥2) between at least two internodes. To confirm the RNA-seq results, the expression of ten differentially expressed genes was determined by qRT-PCR. The RNA-seq and qRT-PCR expression values of these ten genes as well as their patterns of expression were highly similar (Additional file 6). As shown in Additional file 7, 202 transcripts were differentially expressed between Int1 and Int2 and 854 were differentially expressed between Int3 and Int4. The results indicate that Int1 and Int2 are developmentally similar and differ from the more elongated internodes Int3 and Int4.

### Cluster analysis of differentially expressed genes

**Fig. 4:**
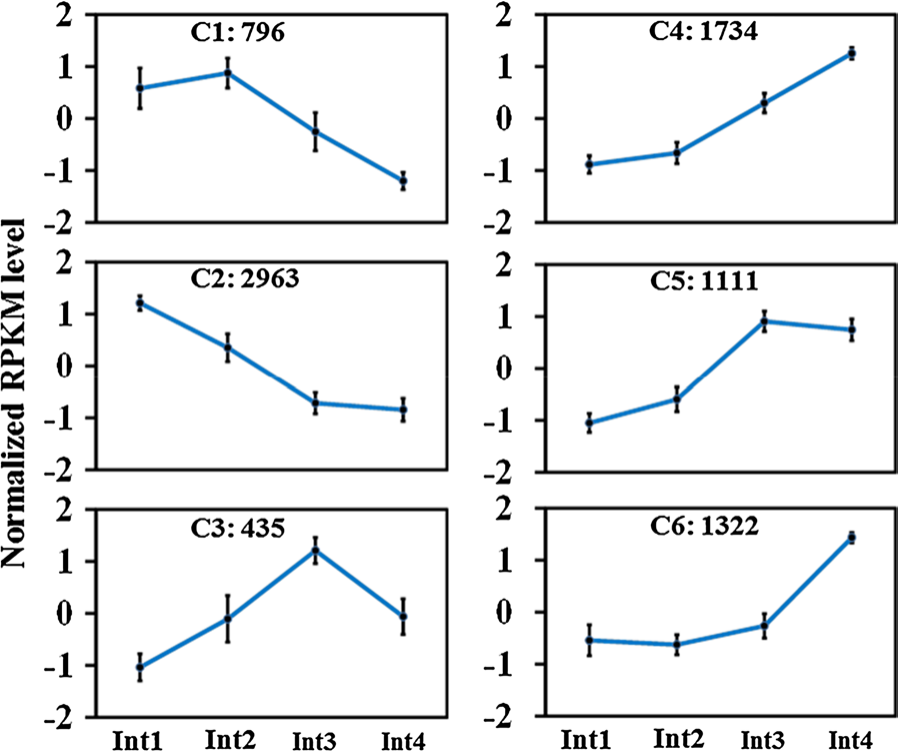
K-means clustering of genes differentially expressed between four sub-apical internodes of bioenergy sorghum inbred R.07020. Genes differentially expressed between at least any two of the four successive sub-apical internodes (Int1–Int4) were grouped into six clusters. Int1 is the youngest sub-apical and Int4 is the oldest internode. Expression level of each gene/transcript in RPKM was normalized prior clustering. Data are mean RPKM of three biological replicates. *Bars* represent standard deviation (SD)

The 8361 differentially expressed transcripts were grouped into six clusters (Fig. 4). Clusters 1 and 2 consist of genes expressed at a higher level in Int1 and Int2. Clusters 4 and 5 include genes expressed at a higher level in Int3 and Int4. The peak expression of genes in Cluster 3 occurred in Int3. Genes in Cluster 6 were expressed at low level in Int1, 2, and 3 and at higher levels in Int4.

The differentially expressed genes were annotated and functionally categorized using the MapMan software (Additional file 8). Functional categorization of the six clusters is shown in Additional files 9 and 10. Most of the genes in Cluster 2 that highly expressed in Int1 and Int2 encode transcription factors (RNA) and genes that function in DNA and protein synthesis, cell division, and development. Most of the genes in Clusters 4, 5, and 6 also encode transcription factors (RNA) and proteins that function in DNA and protein synthesis, cell division and development. A relatively high number of genes involved in hormone-related processes were grouped in Clusters 4 and 6.

To better understand the dynamics of transcriptome changes associated with the developmental progression of vegetative sorghum internode growth, we further analyzed the patterns of expression of genes that function in DNA, protein synthesis, cell division and cell cycle, cell wall biosynthesis, development, and secondary metabolism. We also analyzed the patterns of expression of transcription factors and genes involved in hormone metabolism, signaling and action to identify mechanisms that control the growth of the vegetative internodes from their initiation through elongation.

### Differentially expressed genes involved in cell division and the cell cycle

**Fig. 5:**
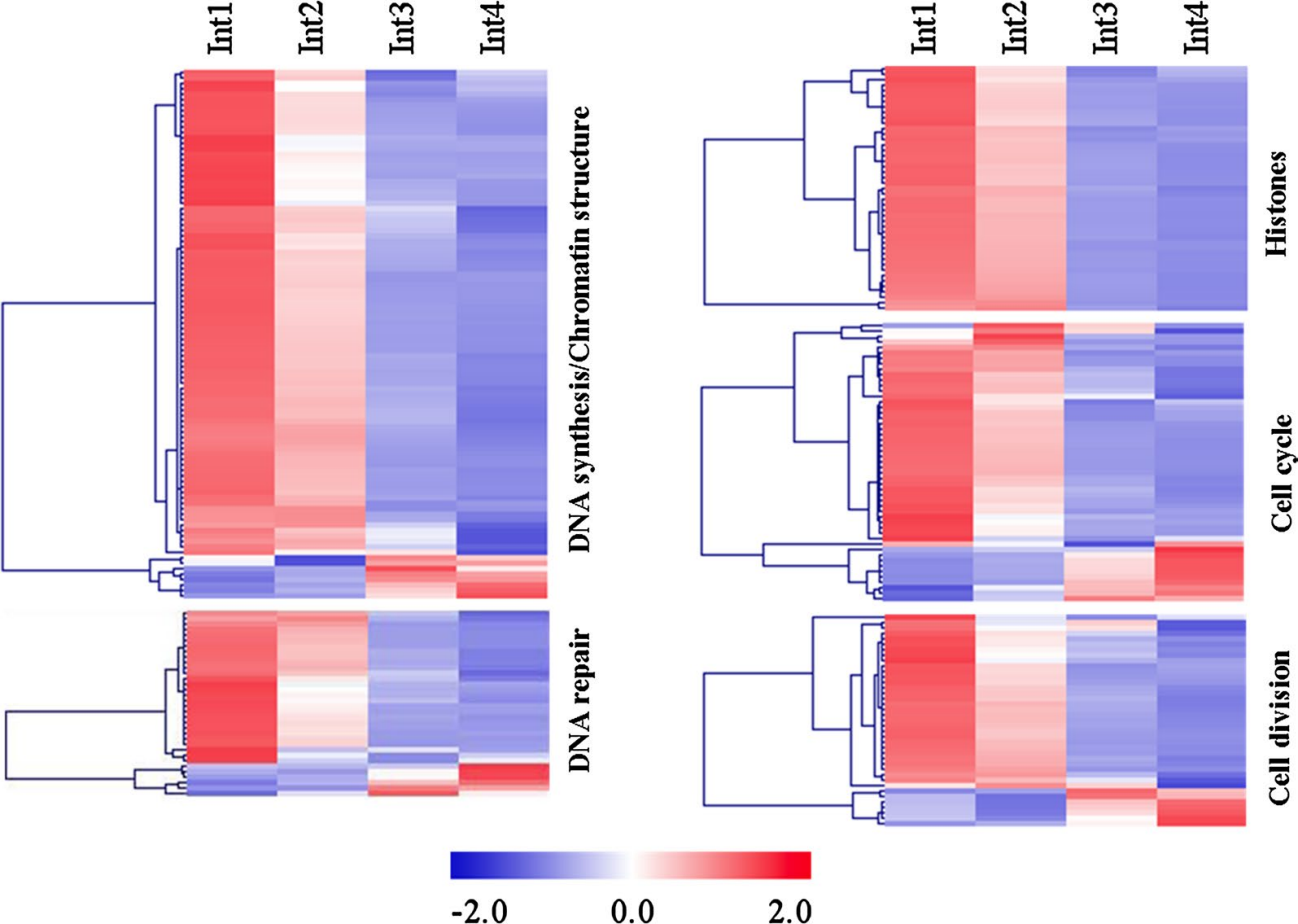
Heatmap of DNA and cell-related genes differentially expressed between four sub-apical internodes of bioenergy sorghum inbred R.07020. Int1 is the youngest sub-apical and Int4 is the oldest internode. Gene expression level in RPKM in each internode was normalized

Plant organ growth involves cell division, elongation, and differentiation, processes that often occur in adjacent spatially separate regions of the growing zones of grass leaves, stems, and roots [41–43]. The first two internodes below the shoot apex (Int1 and Int2) were harvested when 0.7 and 2 cm long, respectively, while Int3 was 4.5 cm, and Int4 had reached its full length of 5 cm. Genes involved in DNA synthesis (histones), repair, and nucleosome assembly were expressed at higher levels in Int1 and Int2 (Fig. 5). Most of the cell cycle genes encoding cyclins and cyclin-dependent protein kinases were expressed at a higher level in Int1/2 (Fig. 5). Genes involved in cell division including the cell division control protein (CDC), RETINOBLASTOMA-RELATED protein (RBR1), and ANAPHASE PROMOTING COMPLEX showed elevated expression in Int1/2.

### Genes involved in cell elongation, cell wall biosynthesis, and secondary metabolism

**Fig. 6:**
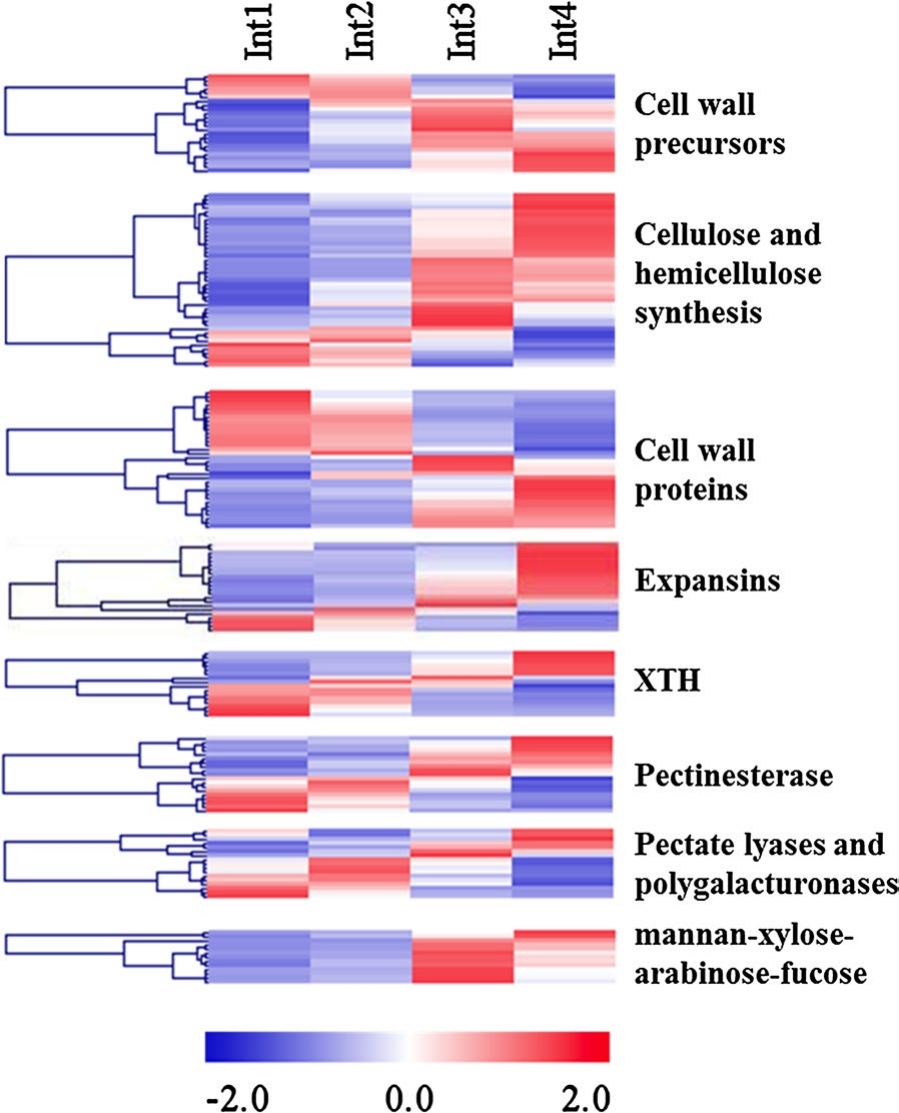
Heatmap of cell wall biosynthesis and modification genes differentially expressed between four sub-apical internodes of bioenergy sorghum inbred R.07020. Int1 is the youngest sub-apical and Int4 is the oldest internode. Gene expression level in RPKM in each internode was normalized

The expression of many genes involved in cell wall biosynthesis increased from Int1/2 to Int3/4 (Fig. 6). However, closer examination of the profiles showed a more complex pattern with subsets of genes involved in cell wall biosynthesis with maximal expression in Int1, Int2, Int3, or Int4. Genes encoding UDP-glucose dehydrogenases (UGD), UDP-glucuronic acid decarboxylase (UXS), and UDP-d-XYLOSE 4-EPIMERASE (MUR4) were expressed at a higher level in Int3/4. These genes catalyze reactions leading to the production of arabinose, galacturonic acid, apiose, xylan, and xyloglucan, constituents of plant cell walls [44–46]. The expression of sorghum genes involved in cellulose (cellulose synthases, *CESA*) and hemicellulose (glucuronoxylan and xyloglucan fucosyltransferase) synthesis was generally higher in Int3/4. About half of the genes encoding cell wall proteins were expressed at higher level in Int1/2 while the other half were expressed at a higher level in Int3/4. Many genes encoding expansins that are involved in cell growth were expressed at higher levels in Int3/4, although several family members were differentially expressed in Int1 and Int2. Most of the genes encoding pectate lyases and polygalacturonases were expressed at a higher level in Int1/2. Some of the genes encoding pectinesterase and xyloglucan endotransglucosylase/hydrolases (XTH) were expressed at a higher level in Int1/2 and others in Int3/4. The patterns of expression of the XTH genes indicate that different members of this gene family are involved in cell wall modification at different stages of internode development. Genes encoding alpha-l-arabinofuranosidase/beta-d-xylosidase were expressed at a higher level in Int3.

**Fig. 7:**
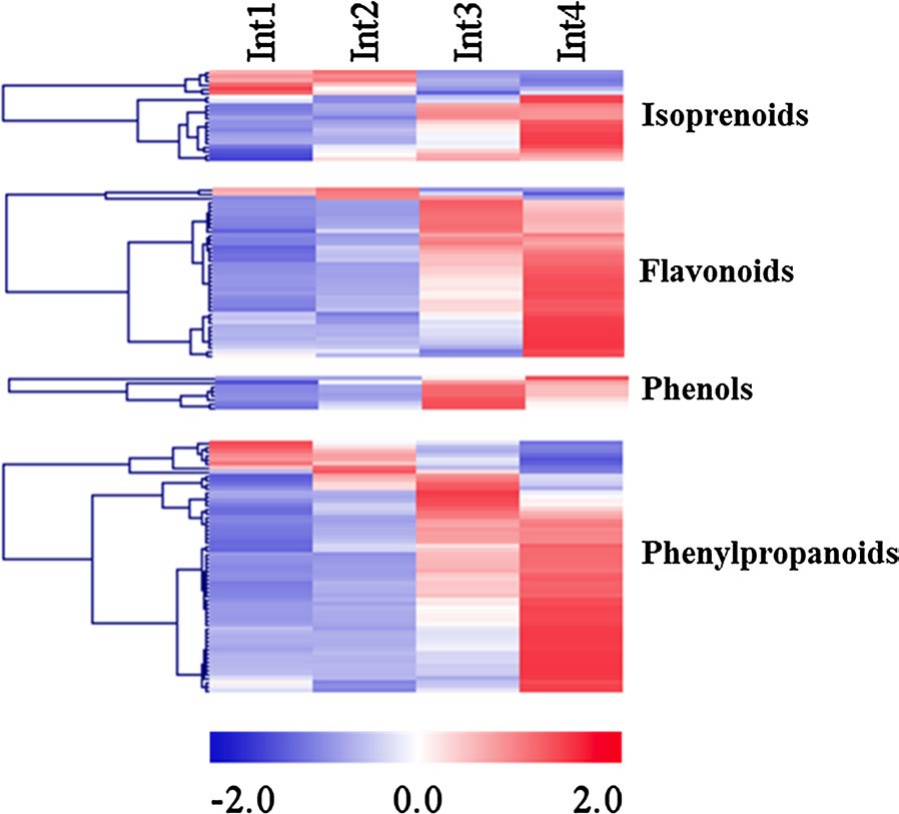
Heatmap of secondary metabolism-related genes differentially expressed between four sub-apical internodes of bioenergy sorghum inbred R.07020. Int1 is the youngest sub-apical and Int4 is the oldest internode. Gene expression level in RPKM in each internode was normalized

Genes annotated as involved in secondary metabolism were generally expressed at a higher level in Int4 (Fig. 7). These genes are involved in the biosynthesis of isoprenoid, flavonoid, phenol, and phenylpropanoids (i.e., lignin). The accumulation of lignin in cell walls reduces cell wall extensibility and increases cell wall strength. Genes in the phenylpropanoid pathway involved in all steps of lignin biosynthesis were differentially expressed during internode development, all showing elevated expression in Int3/4 compared to Int1/2 (Table 1).

**Table 1:**
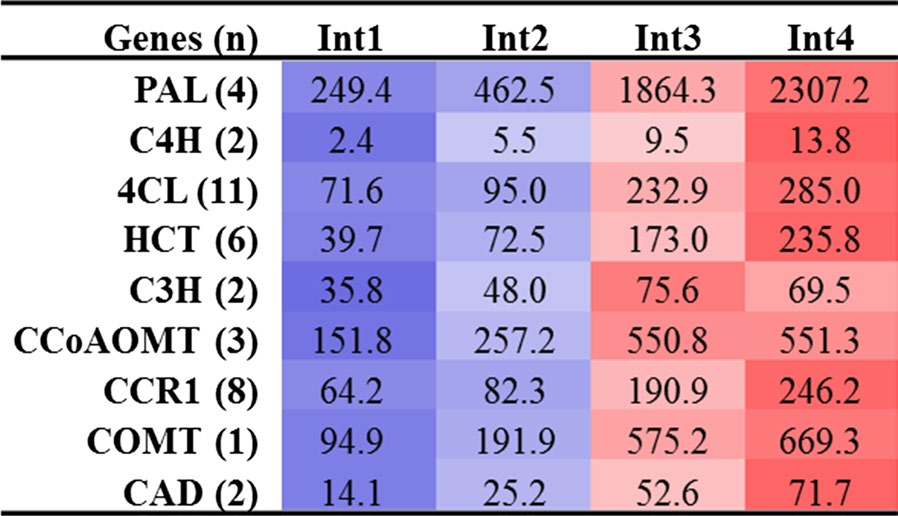
The expression level of lignin biosynthesis genes differentially expressed between four sub-apical internodes of bioenergy sorghum inbred R.07020

Int1 is the youngest internode below the shoot apex and Int4 is the oldest lower internode. Values represent the sum total transcripts in RPKM expressed from a number of genes, except for COMT(1) where the RPKM is from one gene, in the genome shown in parenthesis encoding the same protein. The expression level in each row highlighted with blue is lower and highlighted with red is higher

### Sugar metabolism, signaling, transport, and storage genes

**Fig. 8:**
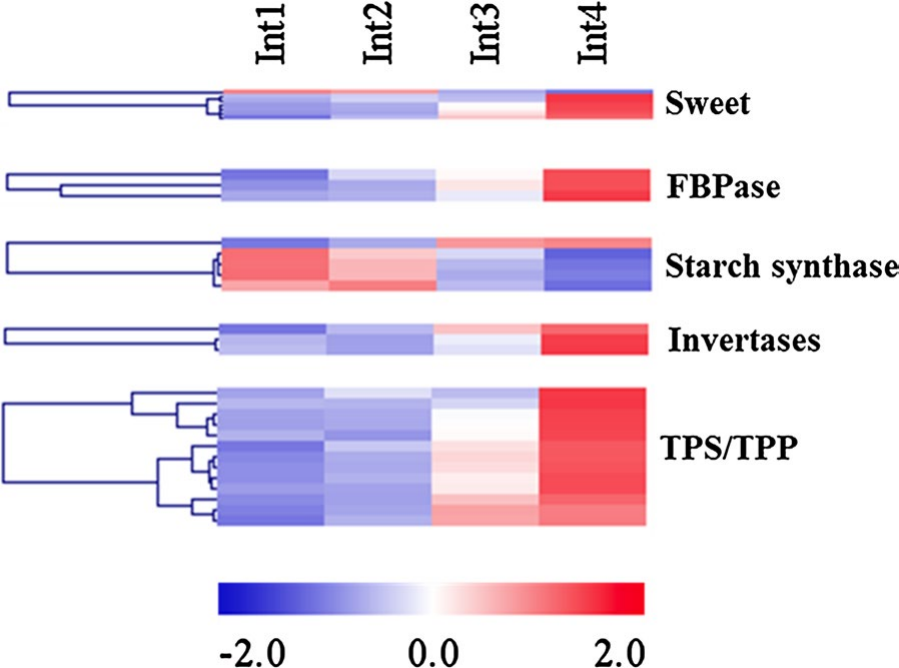
Heatmap of sugar transport, metabolism, and signaling genes differentially expressed between four sub-apical internodes of bioenergy sorghum inbred R.07020. Int1 is the youngest sub-apical and Int4 is the oldest internode. Gene expression level in RPKM in each internode was normalized

Differentially expressed genes involved in sugar metabolism, transport, storage, and signaling include *SWEET* genes that are orthologs of *Medicago truncatula N3 (MtN3)* and *SUT2* sucrose transporters, *fructose*-*1,6*-*bisphosphatase* (*FBPase*), starch synthases, cell wall invertases (*CwINVs*), and genes involved in trehalose metabolism (*TPS* and *TPP*) (Fig. 8). Most of the genes encoding starch synthases were expressed at a higher level in Int1 and at a lower level in Int4, whereas the expression of genes encoding SWEET transporters, FBPase, CwINVs, and trehalose metabolism was higher in Int3 and Int4. The results indicate that the capacity for starch synthesis was higher in the younger internodes and for sucrose transport and metabolism increased in Int3 and Int4. TPS1, a gene involved in trehalose synthesis, was expressed threefold higher in Int3/4 and the expression of several genes encoding TPPs increased >50-fold during the course of internode development (Additional file 11).

### Differentially expressed transcription factors

**Fig. 9:**
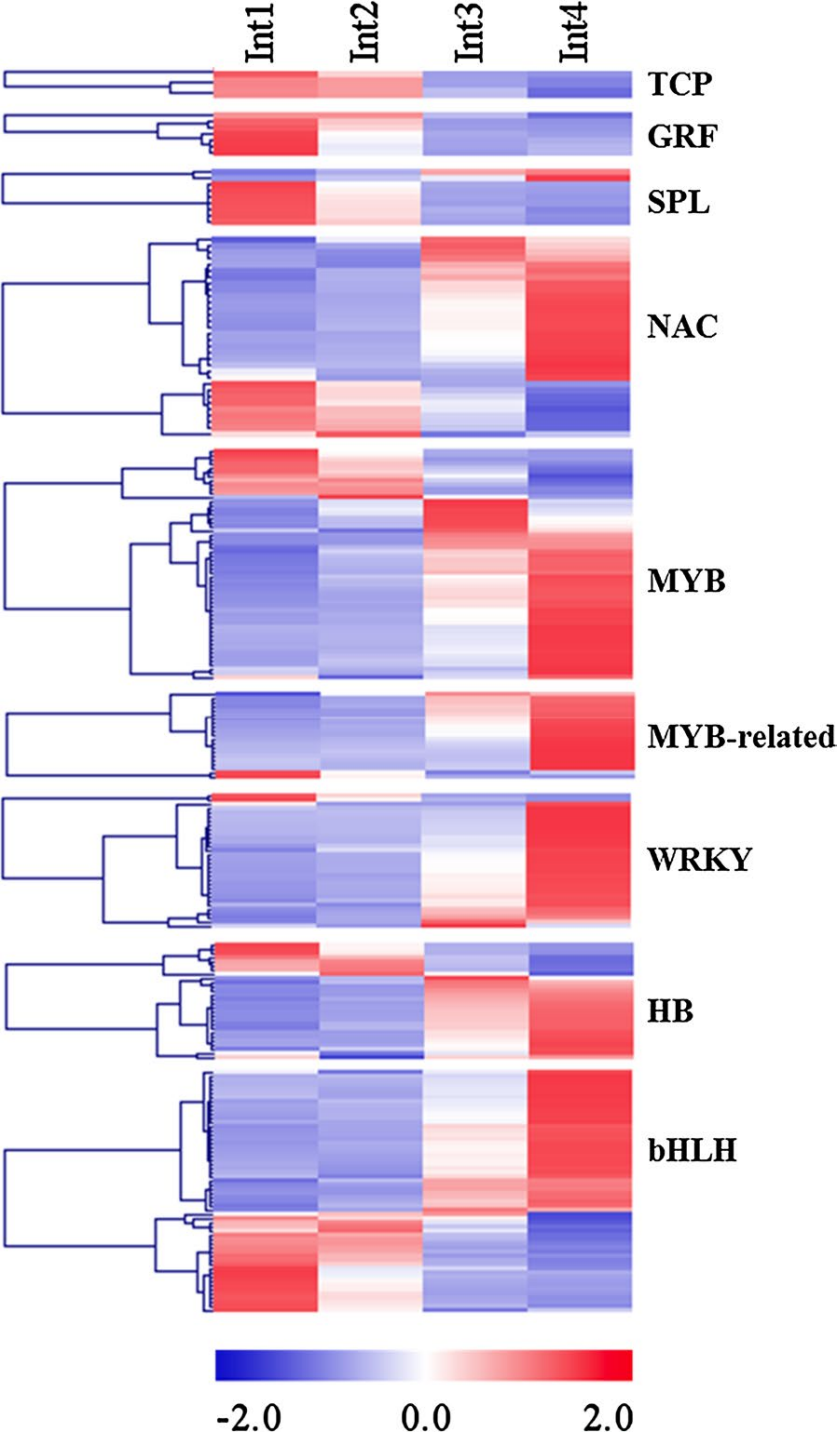
Heatmap of genes encoding transcription factors differentially expressed between four sub-apical internodes of bioenergy sorghum inbred R.07020. Int1 is the youngest sub-apical and Int4 is the oldest internode. Gene expression level in RPKM in each internode was normalized

Approximately 836 transcripts categorized in the MapMan bin RNA, which includes transcription factors (TFs) belonging to diverse families, were differentially expressed during the stages of internode development analyzed (Fig. 9; Additional file 8). The patterns of expression of some of the TFs that function in cell division, elongation, and secondary metabolism are shown in Fig. 9. Three genes encoding *TCP* family transcription factors similar to Arabidopsis *TCP20*, *TCP8*, and *TCP15* were expressed in Int1/2 then down-regulated in Int3/4. TCP20 plays a role in cell proliferation by activating the expression of *CycB* [47] and TCP15 promotes cell proliferation in internodes [48]. Genes encoding GROWTH REGULATING FACTORS (GRFs) were expressed at a high level in Int1 and at decreasing levels during later stages of internode development. The sorghum SQUAMOSA-binding factors that are homologs of Arabidopsis *SPL9* and rice *OsSPL14* were expressed in Int1 then down-regulated during later stages of internode development. *OsSPL14* in rice was found previously to regulate growth, stem length/diameter, tillering, and panicle branching [49, 50].

The NAC transcription factors regulate diverse aspects of plant developmental processes and responses to biotic and abiotic stress [51]. Most of the sorghum NAC domain transcription factors were expressed at a higher level in Int3 and Int4 (Fig. 9) including genes that function in secondary cell wall biosynthesis (*NAC SECONDARY WALL THICKENING PROMOTING FACTOR1*, *NST1*) and ABA biosynthesis (*AtAF1*). NTS1 and its homolog SECONDARY WALL-ASSOCIATED NAC DOMAIN PROTEIN1 (SND1) directly regulate the expression of genes encoding MYB and homeobox protein knotted-1 (KNAT7) transcription factors that regulate secondary cell wall biosynthesis [52]. The expression of two sorghum NAC domain transcription factors that are homologous to the Arabidopsis NAC domain containing protein 36 (ANAC036) was highest in Int4. *ANAC036* overexpressing Arabidopsis lines are dwarf [53].

The MYB, MYB-related, and WRKY family of transcription factors play diverse roles in plant development, secondary cell wall formation and in response to biotic and abiotic stress [54, 55]. Members of these families were up-regulated in Int3/4 (Fig. 9). One of the up-regulated *MYB* genes was annotated as homologous to Arabidopsis *AtMYB42*, a gene involved in secondary cell wall biogenesis [52]. *WRKY*-genes also regulate lignin biosynthesis and play a role in maintaining non-lignified parenchyma cells [56].

Genes that encode HOMEODOMAIN (HB) and Basic Helix-Loop-Helix (bHLH) family of transcription factors are involved in plant development through their actions on meristem maintenance and organogenesis, secondary metabolism, phytochrome signaling, and response to stress. *HOMEODOMAIN* and *bHLH* genes were expressed at a higher level either in Int1/2 or Int3/4 (Fig. 9). Sorghum genes similar to Arabidopsis homeobox knotted-1 (*KNAT3, KNAT4/5* and *KNAT7*) and BEL1-like (*BLH1*, *BLH4* and *BLH6*) showed increased expression in Int3/4. Knotted-1 and BEL1-like homeodomain transcription factors repress secondary wall formation in Arabidopsis and Populus [57–59]. The expression of a sorghum *bHLH* gene weakly similar to the Arabidopsis *PHYTOCHROME INTERACTING FACTOR 3*-*like* was low in Int1/2 and up-regulated in Int3/Int4. In Arabidopsis, PIF3 regulates hypocotyl length and chloroplast development in response to light [60]. Other bHLH transcription factors up-regulated in Int3/4 include a gene similar to the Arabidopsis *BES1*-interacting Myc-like protein 2 (*BIM2*), and a gene annotated as similar to the Arabidopsis *CRYPTOCHROME*-*INTERACTING BASIC*-*HELIX*-*LOOP*-*HELIX 1* (*CIB1*), both involved in light signaling [61, 62].

### Differentially expressed genes involved in hormone metabolism, signaling, and response

**Fig. 10:**
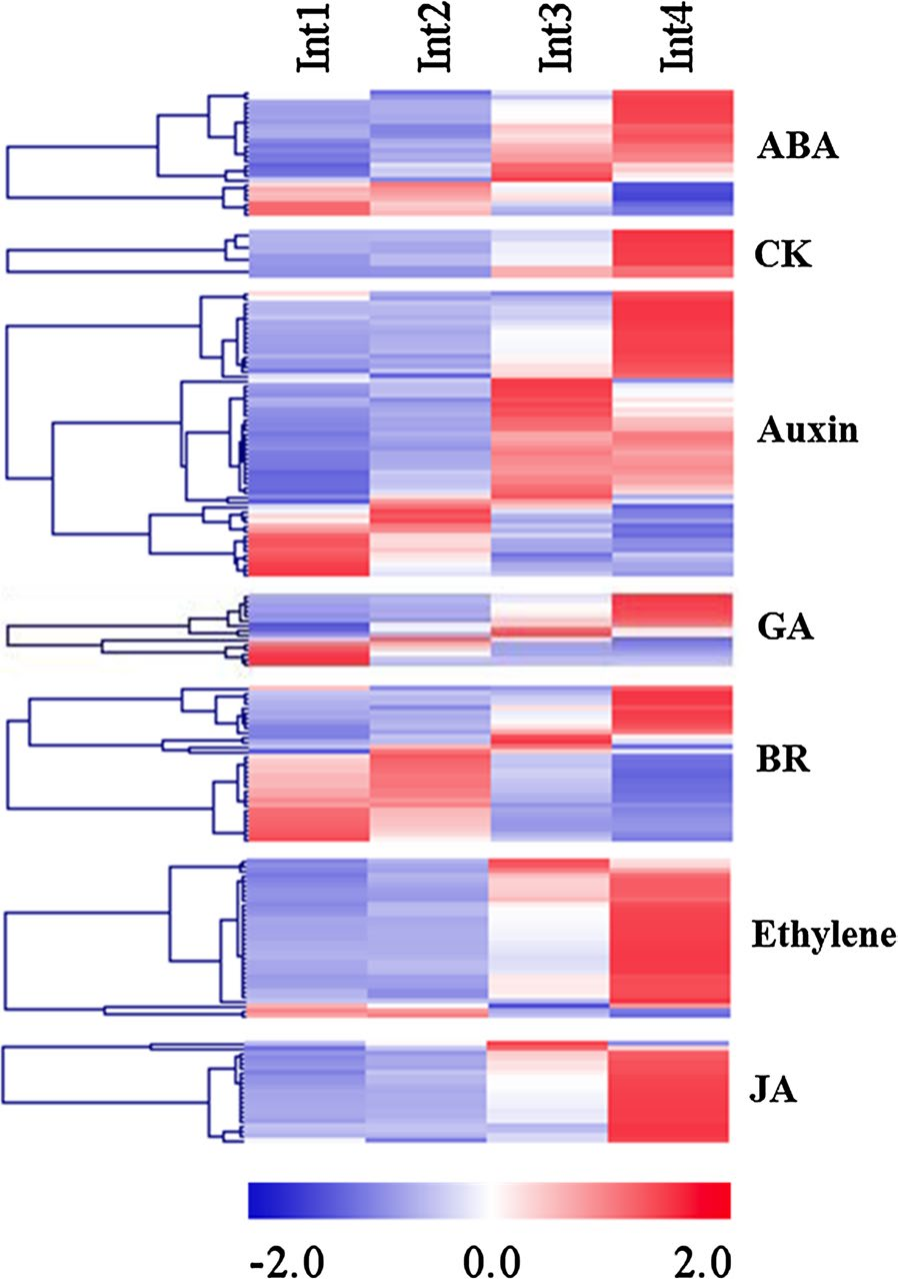
Heatmap of plant hormone biosynthesis, transport, and signaling genes differentially expressed between four sub-apical internodes of bioenergy sorghum inbred R.07020. Int1 is the youngest sub-apical and Int4 is the oldest internode. Gene expression level in RPKM in each internode was normalized

About 190 genes involved in plant hormone metabolism, signaling, and response were differentially expressed during the phase of internode development analyzed in this study (Fig. 10; Additional file 8). Most of the differentially expressed genes involved in ABA, auxin, CK, ethylene, and JA-related genes were expressed at higher levels in Int3/4. BR-related genes were expressed at a higher level in Int1/2 and down-regulated in Int3/4. Some of the key genes that could modify the level or signaling status of the plant hormones are shown in Additional file 12. The up-regulation of genes that encode *NINE*-*CIS*-*EPOXYCAROTENOID DIOXYGENASES* (*NCED3, NCED9*) in Int3/4 indicates an increase in ABA biosynthesis may occur in Int3 and Int4. However, the expression of a gene encoding abscisic aldehyde oxidases (ABA 8′-hydroxylase) that catalyzes ABA turnover was also up-regulated in these internodes. The simultaneous increase in the expression of ABA biosynthesis and catabolism genes could be to limit the activity of ABA to specific tissues or sub-regions of the internode. The expression of a sorghum gene involved in strigolactone (SL) biosynthesis highly similar to the Arabidopsis *CAROTENOID CLEAVAGE DIOXYGENASE7* (*CCD7*) increased in Int4. Increased production of strigolactone in stem internodes could contribute to inhibition of tiller outgrowth as well as other growth responses.

Genes involved in cytokinin biosynthesis were not differentially expressed in Int1–Int4. Instead, sorghum homologs of Arabidopsis cytokinin oxidase/dehydrogenases (*CKX5/6*) that encode enzymes involved in cytokinin catabolism were expressed at higher levels in Int3/4 (Additional file 12). In parallel, expression of two cytokinin responsive AP2/EREBP genes decreased during internode development indicating cytokinin levels and signaling decrease as the internodes elongate and differentiate.

Several genes encoding AUX/IAA factors were differentially expressed at higher levels in Int1/2, while other members of this gene family showed higher expression in Int3/4 (Additional file 8). Similarly, specific members of the SAUR-like, auxin-resistant, and auxin-responsive auxin efflux [*PIN*-*FORMED (PIN)*] gene families showed maximal expression in either Int1/2 or Int3/4. The expression of a sorghum gene highly similar to the Arabidopsis auxin receptor *TRANSPORT INHIBITOR RESPONSE1* (*TIR1*) was reduced in Int3/4 relative to Int1/2 (Additional file 12).

Several genes involved in BR biosynthesis such as *DWARF 1* (*DWF1*), *STEROL METHYLTRANSFERASE 1* (*SMT1*) and *STEROL 1* (*DWF7*) and *CYCLOARTENOL SYNTHASE 1* (*CAS1*) were expressed at a higher level in Int1/2 and down-regulated in Int3/4 (Additional file 12). Sorghum genes similar to the Arabidopsis leucine-rich repeat receptor-like kinase *brassinosteroid insensitive1* (*BRI1*) were expressed in Int1/2 and down-regulated in Int3/4. A sorghum gene highly similar to the Arabidopsis *BRASSINOSTEROID INSENSITIVE 3* (*BIN3*, topoisomerase 6 subunit B) was also down-regulated during internode development. The Arabidopsis *bin3* mutant is dwarf most likely because expression of BR-regulated genes required for growth is reduced in the mutant [63]. In addition, a BR-responsive gene encoding Ring-H2 finger protein in Arabidopsis that is down-regulated by BR [64] was up-regulated in Int3/4.

GA is an important regulator of stem and hypocotyl growth and development [65]. A gene involved in GA biosynthesis (*ENT*-*COPALYL DIPHOSPHATE SYNTHETASE 1* (*CPS1*)] was down-regulated while another gene [*ENT*-*KAURENOIC ACID HYDROXYLASE* (*KAO2*)] was up-regulated in Int3/4. A sorghum gene ortholog of the rice GA receptor gene (*OsGID1*) was up-regulated in Int3/4. However, five genes encoding GA2oxidases that deactivate GA were up-regulated in Int3 and Int4 suggesting that GA levels in specific tissues or sub-regions of elongating internodes may be modified by these genes. The expression of GA responsive genes was generally lower in Int3/4 (Additional file 8).

Genes involved in ethylene biosynthesis and signaling were strongly induced during internode development. These include a gene encoding 1-aminocyclopropane-1-carboxylate synthase 6 (*ACS6*) and three genes encoding 1-aminocyclopropane-1-carboxylate oxidase (ACC oxidase) that were up-regulated in Int3/4 (Additional file 12). The expression of genes encoding ethylene response factors involved in ethylene signaling was also up-regulated in Int3/4.

Genes for jasmonate (JA) biosynthesis including lipoxygenase (*LOX*), allene oxide synthase (*AOS*), 12-oxophytodienoate reductase (*OPDA*-*REDUCTASE 3*), and jasmonic acid carboxyl methyltransferase (*JMT*) were up-regulated in Int3 and Int4 (Fig. 10; Additional file 12). In addition, genes encoding jasmonate-zim-domain proteins that mediate JA-responses were up-regulated in Int3/4.

## Discussion

The purpose of this study was to document anatomical changes and transcriptome dynamics that occur during vegetative internode development of bioenergy sorghum. Plant organ growth involves cell division, elongation, and differentiation, processes that often occur in spatially separate regions of the growing zones of grass leaves, stems, and roots [41–43]. The development of stem internodes in 60-day-old vegetative sorghum plants was investigated by characterizing the growth, anatomy, and transcriptomes of sub-apical internodes during an early stage of development through full elongation. The first visible sub-apical internode below the dome of tissue enclosing the SAM contained all of the tissue types found in a fully elongated internode. During development cells that comprise the internode became larger in diameter and elongated to varying extents depending on cell type and location within the internode. In fully elongated internodes, cells located towards the center of the stem were larger than those closer to the epidermis. A gradient of lignification was also observed in fully elongated internodes where vascular bundles located in the central region of the stem were more lignified than those located near the periphery of the internode. The retention of small less lignified cells in the stem rind (epidermis and sub-epidermal zone) may allow continued growth in stem diameter during vegetative development of energy sorghum that can continue for more than 150 days [66]. While most genes encoding cyclins were down-regulated during internode development, a small group of cyclin genes showed increased expression in mature internodes. This also indicates that the potential for cell division is retained in mature vegetative sorghum stem internodes that may contribute to increases in stem diameter. The expression of genes involved in secondary cell wall formation remained high in fully elongated internodes for at least 20 days in the sweet sorghum Della (McKinley et al. [27]). Therefore, once internodes stop elongating, stem cells continue to differentiate in part by increasing the thickness of secondary cell walls of xylem and sclerenchyma cells of the vascular system and fibers that provide stem strength.

Stem internodes derived from the SAM sequentially traverse phases of development involving cell division, cell elongation, and cell differentiation in spatially distinct regions of the apical growing zone. Growing zones of roots [41], leaves [42], and stems [26] of many plants are organized in a similar manner. Microscopic analysis of sorghum stem internodes revealed the presence of dividing cells in the two youngest sub-apical internodes (Int1/2) that were not observed in mid-internode cross sections of the more elongated internodes 3 and 4. Consistent with the microscopic results, RNA-seq analysis identified a large group of genes involved in cell division and the cell cycle (i.e., cyclins) that were differentially expressed at elevated levels in the two youngest internodes. This indicates that newly formed internodes immediately below the SAM initially increase in size due to cell-division coupled growth prior to the onset of rapid cell and internode elongation that occurs during the transition from Int2 to Int3 (Fig. 2). This developmental transition involves a change from delocalized cell-division coupled growth (Int1/2), to internodes (Int3) that exhibit polarized growth resulting in the accumulation of elongated cells in the more mature lower internodes (Fig. 2) [25, 26, 67]. Martin et al. identified a region of meristematic zone in the basal section of a rapidly expanding internode of *Setaria viridis* [26]. Bosch et al. compared gene expression in elongating and non-elongating (mature) maize internodes [24]. Although the lower 1-cm meristematic zone of both elongating and non-elongating internodes was not included in the samples, cell cycle and DNA synthesis genes were expressed in the younger elongating maize internode. It appears that cell division in young grass stem internodes is dispersed prior to elongation, and an intercalary meristematic region is retained in the basal section of elongating internodes.

Genes that regulate the size of meristems, the duration of cell-division coupled growth, and the transition between regions of cell division and cell elongation are of interest because of their impact on root, stem, or leaf size. The expression of several genes encoding class I TCP-transcription factors, GRF-factors, and squamosa promoter binding factors was at high levels in Int1/2 and at lower levels in Int3/4. Class I TCP-transcription factors regulate cyclin gene expression and cell proliferation [68, 69]. The patterns of expression of the class I TCP gene are consistent with higher levels of mitotically active cells in Int1/2 and lower levels in Int3/4 (Fig. 1). GRF-factors modulate many aspects of plant growth, and genes encoding these proteins are differentially expressed in zones of cell proliferation [70]. Lower expression of three GRF-genes reduces leaf size while over-expression increased the leaf size by increasing leaf number [71]. The GRF-genes that are differentially expressed in young sorghum internodes with elevated rates of cell division could regulate internode size in a similar manner. In rice, *OsSPL14* inhibits tillering and increases petiole elongation, stem length and diameter, and panicle branching [49, 50]. Therefore, it is possible that elevated expression of the *OsSPL14*-like gene in Int1 enhances cell proliferation prior internode elongation. Further analysis will be required to clarify the function of class I TCP-transcription factors, GRF-factors, and Squamosa promoter binding factors during sorghum internode development.

Suspension of cell division in the more differentiated Int3/4 is accompanied by an increase in the expression of genes associated with secondary metabolism. In addition, genes involved in sucrose metabolism, transport, and signaling including *SWEET* and *SUT2* sucrose transporters, *fructose*-*1,6*-*bisphosphatase* (*FBPase*), starch synthases, cell wall invertases (*CwINVs*) and genes involved in trehalose metabolism (*TPS* and *TPP*) were up-regulated in Int3/4. The results indicate enhanced metabolic activity in the more differentiated internodes. Interestingly, the expression of starch synthesis genes was up-regulated in the growing internodes. It is possible that excess sucrose in the younger internodes is stored temporarily as starch to be used for growth in the absence of sucrose supply from photosynthetic leaves during the night [72, 73].

The transition from Int2 to Int3 was associated with a significant increase in internode elongation due in part to an increase in the length of cells. Several genes encoding expansins and XTHs were coordinately up-regulated during the transition to internode elongation that may contribute to this growth response (Fig. 6). Genes encoding bHLH factors regulate rates of cell elongation by integrating signals from phytochrome (SAS), GA, and BR [74–77]. Genes encoding bHLH factors showed higher expression in Int3/4 compared to Int1/2. In particular, the expression of the sorghum gene annotated as *BIM1* increased during this phase of internode development. BIM1 is part of a module that includes BES1/PAR1 that regulates cell elongation [77]. *BIM* mutants (*bim1/2/3*) in Arabidopsis have shorter hypocotyls when grown in darkness [61]. BIM together with a second regulatory module involving PAR1/COP1 and BBX21 regulate shade avoidance responses [78]. Therefore, it was interesting that a sorghum gene encoding BBX21 was expressed at higher levels in Int3/4 (Additional file 8).

The cessation of cell elongation is associated with increased synthesis of lignified secondary cell walls. While growth of all internodes requires synthesis of cellulose, deposition of secondary cell walls in grasses is associated with increased expression of *CESA4/7/9* and genes encoding enzymes in the lignin pathway [79]. Transcriptome analysis showed that *CESA*-genes involved in cell wall biosynthesis were expressed in all of the internodes analyzed and at a higher level in Int3/4. The vascular bundles in the central region of the first three internodes mainly stained with alcian blue indicative of primary cell walls. In contrast, vascular bundles of the fourth internode stained strongly with safranin, indicating the accumulation of lignin containing secondary cell walls. Genes encoding enzymes involved in lignin biosynthesis were induced in parallel with increased lignin staining. The regulatory network that mediates secondary cell wall biosynthesis is complex and enriched in genes encoding NAC and MYB factors that are organized in a hierarchical regulatory structure [80]. Genes encoding MYB and NAC show peak expression in Int4. Genes encoding WRKY transcription factors were also up-regulated in Int3/4, and some of these genes may function to prevent secondary cell wall formation on parenchyma cells of the stem [81]. A sorghum gene annotated as a homolog of homeobox protein knotted-1 (*KNAT7*), a repressor of secondary cell wall formation [57], was expressed at higher levels in Int3/4 compared to Int1/2. KNAT7 represses secondary cell wall formation through interactions with BEL1-like transcription factors [58, 59]. Sorghum genes similar to *BEL1*-*like* were up-regulated in Int3/4 indicating that the KNAT7/BEL1-like regulatory module may inhibit secondary cell wall formation in pith parenchyma or reduce secondary cell wall deposition in phloem tissues.

The role of plant hormones in regulating internode growth has been demonstrated using mutants defective in internode elongation. In general, mutants with disruptions in auxin, GA or BR biosynthesis, signaling, or transport exhibit dwarf or small leaf phenotypes. In this study, we focused on the identification of genes involved in hormone biosynthesis, metabolism, signaling, and action that are expressed in internodes during development (Fig. 10). Interestingly, genes involved in cytokinin and auxin biosynthesis were not differentially expressed during the phase of internode development analyzed, and genes involved in BR synthesis were down-regulated in Int3/4. However, genes encoding proteins that transport or metabolize these hormones were differentially expressed during internode development. For example, two genes encoding proteins that deactivate cytokinin (*CKX5* and *CKX6*) showed increased expression during internode development in parallel with decreased expression of two genes encoding cytokinin response factors indicating a reduction in CK level may occur during internode development. In addition, genes encoding GA2oxidases that deactivate GA were up-regulated during internode development, possibly contributing to polar growth across Int3 and/or the cessation of internode elongation. Genes encoding auxin response factors (AUX/IAA) and other auxin-regulated genes were differentially expressed in a highly complex way with different family members showing peak expression at different stages of internode development. This complexity is not surprising given the numerous roles auxin plays in growth and differentiation, the highly articulated auxin transport and regulatory network, and cell-specific actions of this hormone [82, 83]. Interestingly, the expression of *TIR1*, a gene that is involved in auxin signaling, decreased during internode development. Therefore, it appears that the level, distribution and signaling of auxin, cytokinin, and GA are specified in a manner that regulates internode growth and development.

The hormones ethylene, ABA, and JA modulate growth and play a role in plant responses to abiotic stress, and pathogens/insects. Transcriptome analysis showed that genes involved in the synthesis and action of all three hormones increased during internode development. For example, expression of *ACS6* was induced in Int2 and further up-regulated in Int3 and Int4. Ethylene is known to inhibit the cell cycle through down-regulation of cyclin gene expression [84]. Therefore, the initial induction of ethylene synthesis may contribute to the decrease in cell division observed between Int2 and Int3. Ethylene also promotes internode elongation in light grown plants and could therefore contribute to internode elongation [84, 85]. However, a high level of ethylene inhibits growth and stimulates cell differentiation [85, 86]. Therefore, as previously proposed, ethylene may initially stimulate internode elongation and then inhibit growth as ethylene levels rise in more fully elongated internodes. A large number of genes that mediate ethylene responses are induced during internode development indicating that this hormone may be playing several different roles. Additional analyses with greater spatial and temporal resolution will be helpful in defining the role of ethylene in vegetative phase internode development.

Jasmonate (JA) mediates the induction of genes involved in insect defense, and together with ethylene, modulates plant responses to abiotic stress [87]. Genes involved in JA biosynthesis such as lipoxygenases (*LOX*), allene oxide synthase (*AOS*), 12-oxophytodienoate reductase (*OPDA*-*REDUCTASE 3*), and jasmonic acid carboxyl methyltransferase (*JMT*) were up-regulated in Int3 and Int4. In addition, a large number of JAZ-genes (jasmonate ZIM domain proteins) were induced with a similar time course. JA is involved in many aspects of plant development including reproductive development, anthocyanin accumulation, trichome development, and leaf senescence [88–91]. Moreover, JA can have a negative effect on growth through an interaction with DELLA [92]; therefore, JA may modify the extent of internode elongation.

## Conclusions

Shoot biomass in bioenergy sorghum is preferentially accumulated in stem internodes. Stem internodes are formed during the vegetative phase and their length is modulated by genetic, developmental, and environmental signals. Microscopic and gene expression analyses demonstrate that the molecular basis of internode growth and development can be identified in the C4 grass sorghum through analysis of a series of sub-apical internodes that are at various stages of development. In general, genes associated with cell division are up-regulated in newly formed internodes immediately below the shoot apical meristem, while genes involved in secondary wall formation and lignification are up-regulated in internodes as they reach full elongation. Differential expression of numerous genes that modify hormone synthesis and signaling along this developmental gradient indicates that plant hormones play an important role in coordinating internode growth and development. Transcription factors that are differentially expressed during the different stages of vegetative internode growth could be targets of developmental and environmental signals that modulate internode length and girth. The current study provides a baseline of information on changes in stem anatomy and gene expression that occur during development of internodes in vegetative sorghum plants. The molecular basis of increased internode growth induced by shading is the subject of a follow-on study.

## Additional files

**Additional file 1.** Primers used in gene expression analysis by qPCR to validate RNA-seq results.

**Additional file 2.** Vegetative internodes of bioenergy sorghum inbred R.07020 elongate in response to shade and short days.

**Additional file 3.** Total and percentage of mapped and unmapped RNA-seq reads.

**Additional file 4.** Quality control using principal component analysis of RNA-seq data generated from successive sub-apical internodes.

**Additional file 5.** The mean RPKM level of transcripts specifically expressed in one of the four successive sub-apical vegetative internodes.

**Additional file 6.** Validation of RNA-seq results by qPCR. Expression relative to the level in Int1.

**Additional file 7.** The number of differentially expressed transcripts between any two successive sub-apical internodes.

**Additional file 8.** MapMan functional categorization of genes differentially expressed transcripts between any two of four successive sub-apical internodes.

**Additional file 9.** MapMan classification of differentially expressed transcripts between four successive sub-apical internodes grouped in six clusters.

**Additional file 10.** MapMan functional groups with at least ten differentially expressed genes in one of the six clusters.

**Additional file 11.** The expression level in RPKM of trehalose biosynthesis genes in four sub-apical internodes genes between any two of the four successive sub-apical internodes.

**Additional file 12.** Plant hormone metabolism and signaling genes differentially expressed between four sub-apical internodes of bioenergy sorghum inbred R.07020.

## Abbreviations

ARFs: auxin response factors
TIR: transport inhibitor response1
ABA: abscisic acid
SL: strigolactones
CK: cytokinin
GA: gibberellic acid
BR: brassinosteroid
JA: jasmonic acid
RPKM: reads per kilobase of transcript per million mapped reads
PAL: phenylalanine ammonia-lyase
C4H: cinnamate-4-hydroxylase
4CL: 4-coumarate:CoA ligase
HCT: hydroxycinnamoyl-coenzyme A shikimate/quinate hydroxycinnamoyltransferase
C3H: coumarate 3-hydroxylase
CCoAOMT1: caffeoyl coenzyme A *O*-methyltransferase 1
CCR1: cinnamoyl CoA reductase
COMT: caffeic acid/5-hydroxyferulic acid *O*-methyltransferase
CAD: cinnamyl alcohol dehydrogenase
