# Supplementary material for: Dynamics of gene expression during development and expansion of vegetative stem internodes of bioenergy sorghum
Source: Biotechnol Biofuels. 2017 Jun 21;10:159. doi: 10.1186/s13068-017-0848-3 (PMC5480195; doi:10.1186/s13068-017-0848-3)
Supplement: Supplementary file 1 — Additional file 1. Primers used in gene expression analysis by qPCR to validate RNA-seq results. [file 13068_2017_848_MOESM1_ESM.pptx]

## Slide 1
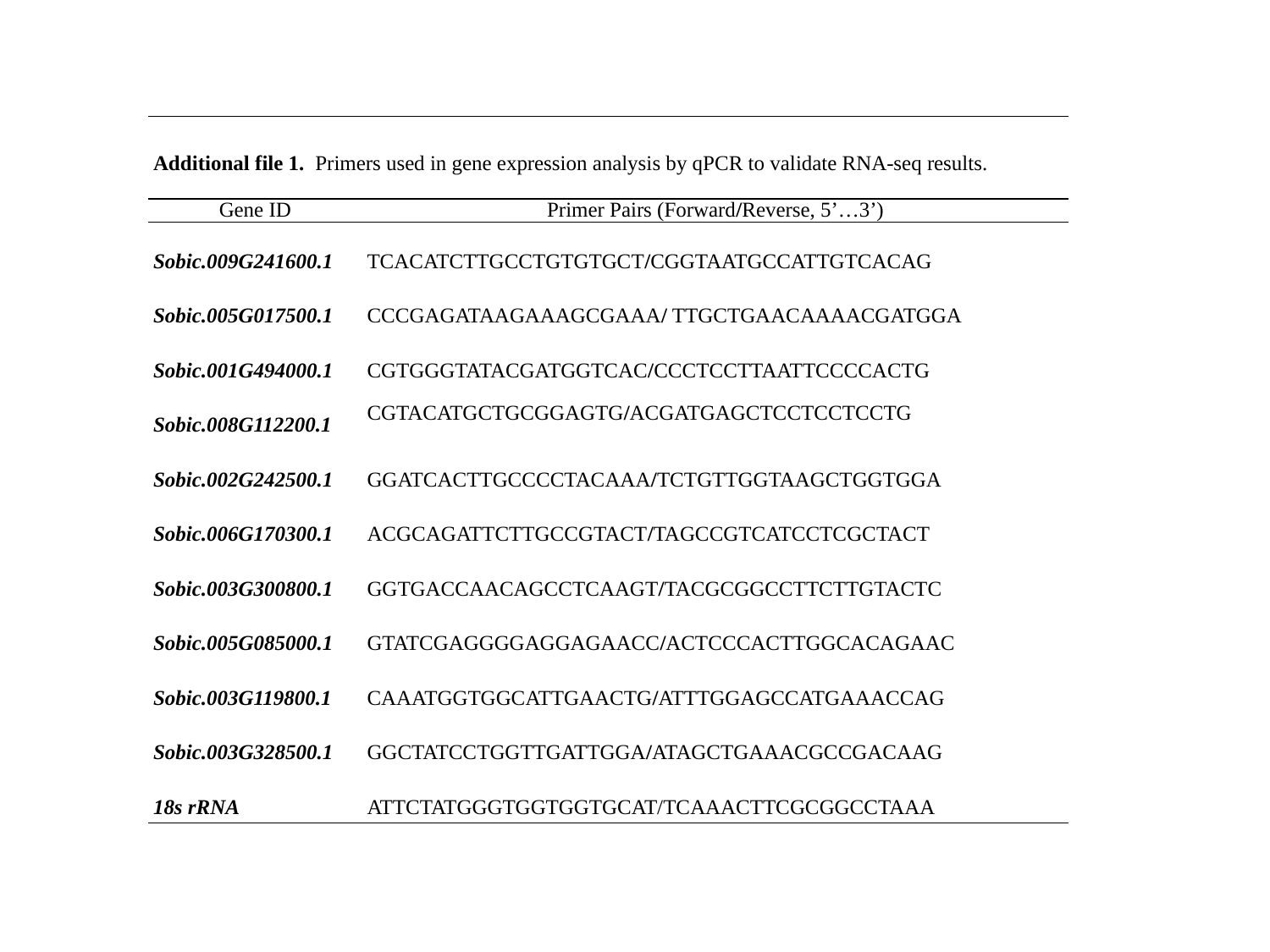

| Additional file 1. Primers used in gene expression analysis by qPCR to validate RNA-seq results. | |
| --- | --- |
| Gene ID | Primer Pairs (Forward/Reverse, 5’…3’) |
| Sobic.009G241600.1 | TCACATCTTGCCTGTGTGCT/CGGTAATGCCATTGTCACAG |
| Sobic.005G017500.1 | CCCGAGATAAGAAAGCGAAA/ TTGCTGAACAAAACGATGGA |
| Sobic.001G494000.1 | CGTGGGTATACGATGGTCAC/CCCTCCTTAATTCCCCACTG |
| Sobic.008G112200.1 | CGTACATGCTGCGGAGTG/ACGATGAGCTCCTCCTCCTG |
| Sobic.002G242500.1 | GGATCACTTGCCCCTACAAA/TCTGTTGGTAAGCTGGTGGA |
| Sobic.006G170300.1 | ACGCAGATTCTTGCCGTACT/TAGCCGTCATCCTCGCTACT |
| Sobic.003G300800.1 | GGTGACCAACAGCCTCAAGT/TACGCGGCCTTCTTGTACTC |
| Sobic.005G085000.1 | GTATCGAGGGGAGGAGAACC/ACTCCCACTTGGCACAGAAC |
| Sobic.003G119800.1 | CAAATGGTGGCATTGAACTG/ATTTGGAGCCATGAAACCAG |
| Sobic.003G328500.1 | GGCTATCCTGGTTGATTGGA/ATAGCTGAAACGCCGACAAG |
| 18s rRNA | ATTCTATGGGTGGTGGTGCAT/TCAAACTTCGCGGCCTAAA |
