# Supplementary material for: Dynamics of gene expression during development and expansion of vegetative stem internodes of bioenergy sorghum
Source: Biotechnol Biofuels. 2017 Jun 21;10:159. doi: 10.1186/s13068-017-0848-3 (PMC5480195; doi:10.1186/s13068-017-0848-3)
Supplement: Supplementary file 2 — Additional file 2. Vegetative internodes of bioenergy sorghum inbred R.07020 elongate in response to shade and short days. [file 13068_2017_848_MOESM2_ESM.pptx]

## Slide 1
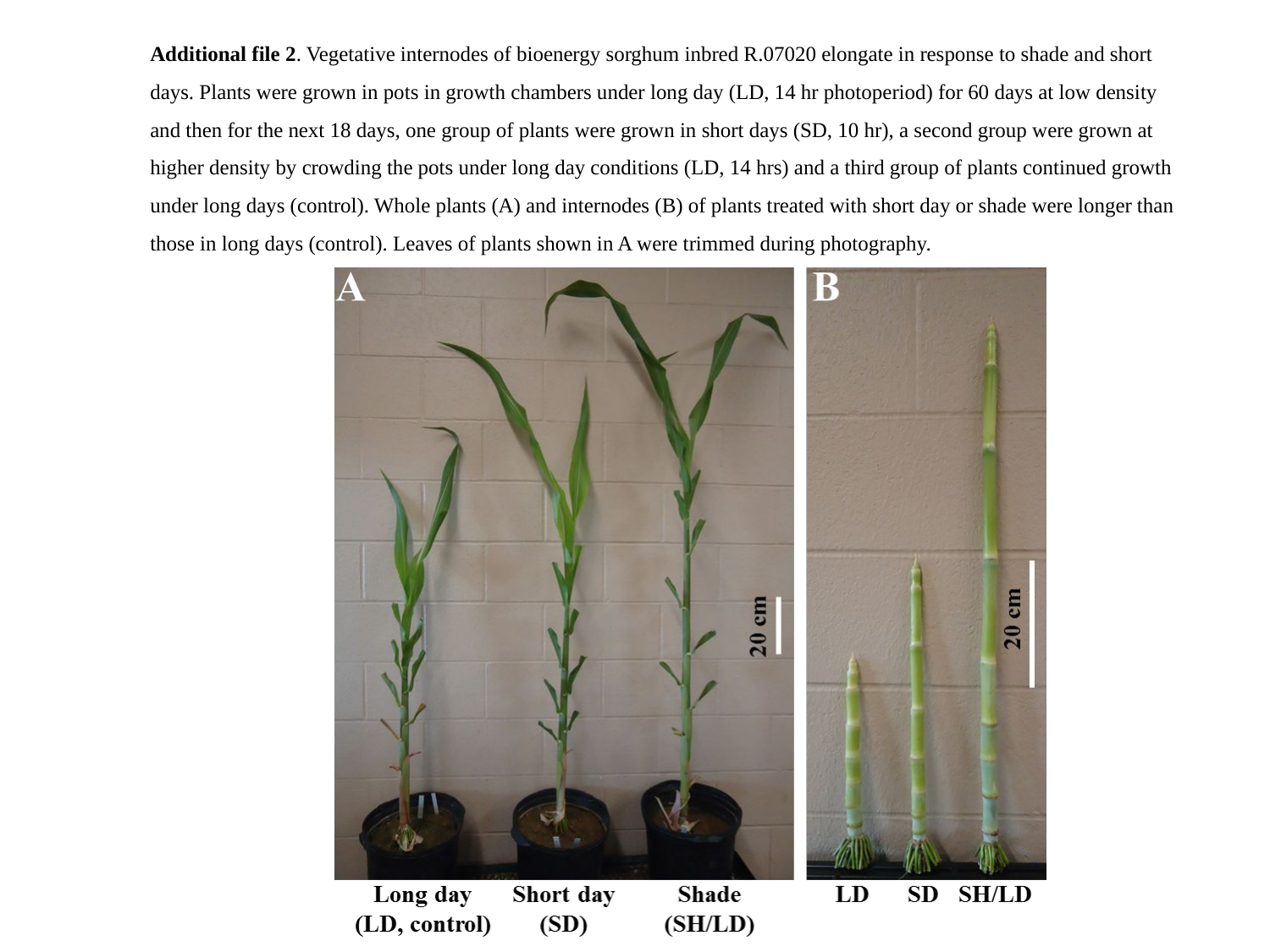

Additional file 2. Vegetative internodes of bioenergy sorghum inbred R.07020 elongate in response to shade and short days. Plants were grown in pots in growth chambers under long day (LD, 14 hr photoperiod) for 60 days at low density and then for the next 18 days, one group of plants were grown in short days (SD, 10 hr), a second group were grown at higher density by crowding the pots under long day conditions (LD, 14 hrs) and a third group of plants continued growth under long days (control). Whole plants (A) and internodes (B) of plants treated with short day or shade were longer than those in long days (control). Leaves of plants shown in A were trimmed during photography.
