# Supplementary material for: Dynamics of gene expression during development and expansion of vegetative stem internodes of bioenergy sorghum
Source: Biotechnol Biofuels. 2017 Jun 21;10:159. doi: 10.1186/s13068-017-0848-3 (PMC5480195; doi:10.1186/s13068-017-0848-3)
Supplement: Supplementary file 3 — Additional file 3. Total and percentage of mapped and unmapped RNA-seq reads. [file 13068_2017_848_MOESM3_ESM.pptx]

## Slide 1
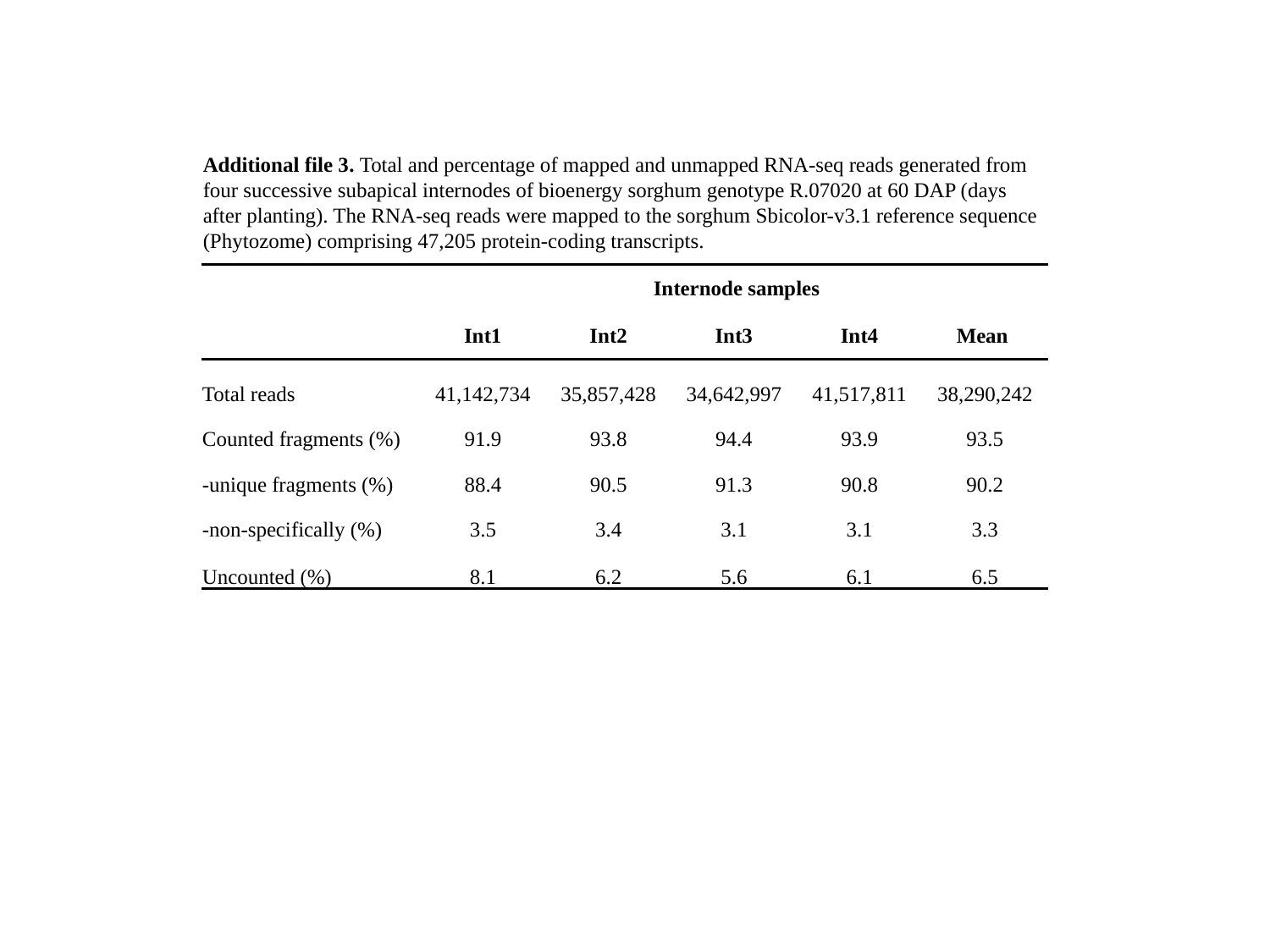

Additional file 3. Total and percentage of mapped and unmapped RNA-seq reads generated from four successive subapical internodes of bioenergy sorghum genotype R.07020 at 60 DAP (days after planting). The RNA-seq reads were mapped to the sorghum Sbicolor-v3.1 reference sequence (Phytozome) comprising 47,205 protein-coding transcripts.
| | Internode samples | | | | |
| --- | --- | --- | --- | --- | --- |
| | Int1 | Int2 | Int3 | Int4 | Mean |
| Total reads | 41,142,734 | 35,857,428 | 34,642,997 | 41,517,811 | 38,290,242 |
| Counted fragments (%) | 91.9 | 93.8 | 94.4 | 93.9 | 93.5 |
| -unique fragments (%) | 88.4 | 90.5 | 91.3 | 90.8 | 90.2 |
| -non-specifically (%) | 3.5 | 3.4 | 3.1 | 3.1 | 3.3 |
| Uncounted (%) | 8.1 | 6.2 | 5.6 | 6.1 | 6.5 |
