# Supplementary material for: Dynamics of gene expression during development and expansion of vegetative stem internodes of bioenergy sorghum
Source: Biotechnol Biofuels. 2017 Jun 21;10:159. doi: 10.1186/s13068-017-0848-3 (PMC5480195; doi:10.1186/s13068-017-0848-3)
Supplement: Supplementary file 4 — Additional file 4. Quality control using principal component analysis of RNA-seq data generated from successive sub-apical internodes. [file 13068_2017_848_MOESM4_ESM.pptx]

## Slide 1
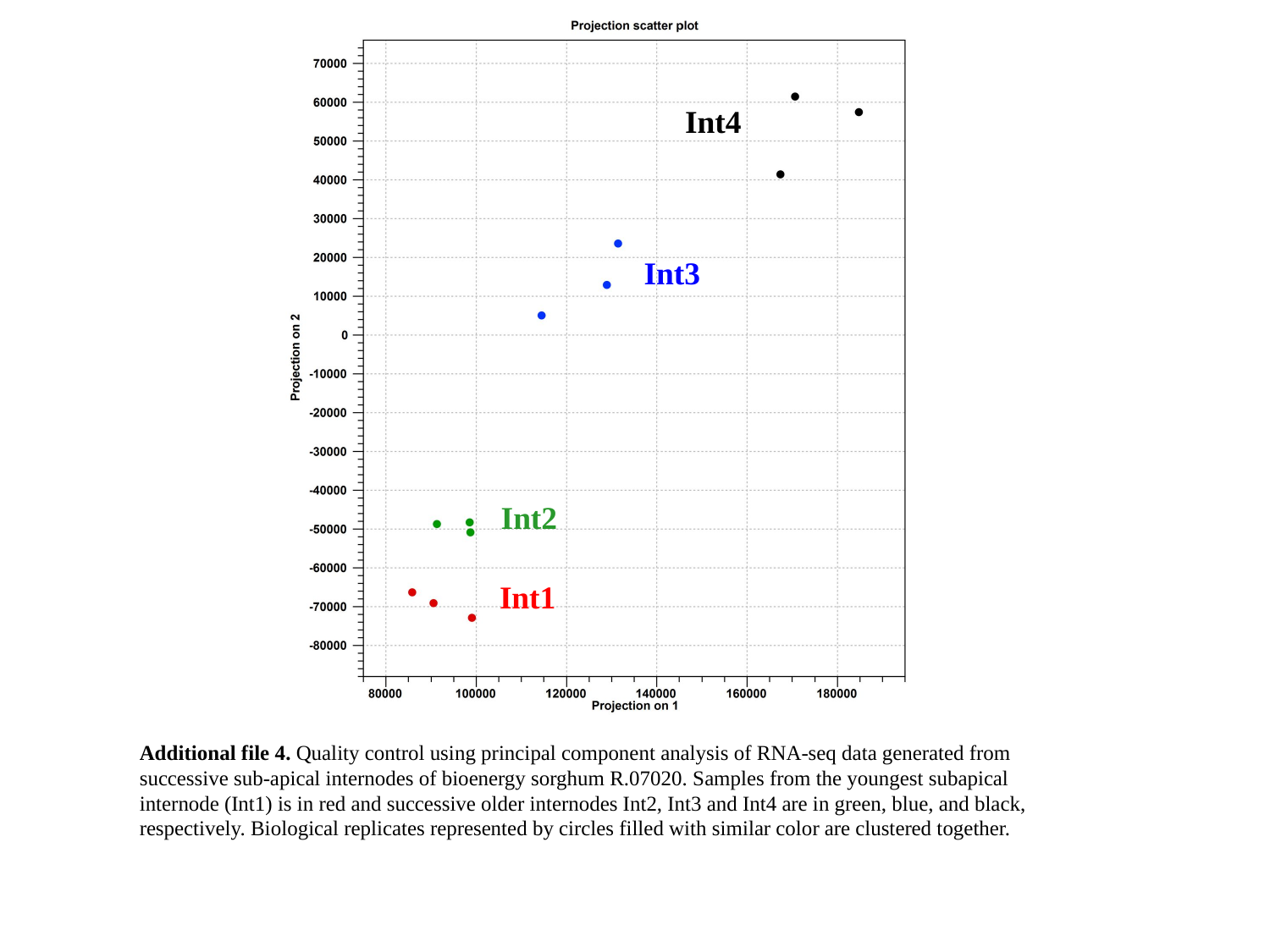

Int4
Int3
Int2
Int1
Additional file 4. Quality control using principal component analysis of RNA-seq data generated from successive sub-apical internodes of bioenergy sorghum R.07020. Samples from the youngest subapical internode (Int1) is in red and successive older internodes Int2, Int3 and Int4 are in green, blue, and black, respectively. Biological replicates represented by circles filled with similar color are clustered together.
