# Supplementary material for: Dynamics of gene expression during development and expansion of vegetative stem internodes of bioenergy sorghum
Source: Biotechnol Biofuels. 2017 Jun 21;10:159. doi: 10.1186/s13068-017-0848-3 (PMC5480195; doi:10.1186/s13068-017-0848-3)
Supplement: Supplementary file 5 — Additional file 5. The mean RPKM level of transcripts specifically expressed in one of the four successive sub-apical vegetative internodes. [file 13068_2017_848_MOESM5_ESM.pptx]

## Slide 1
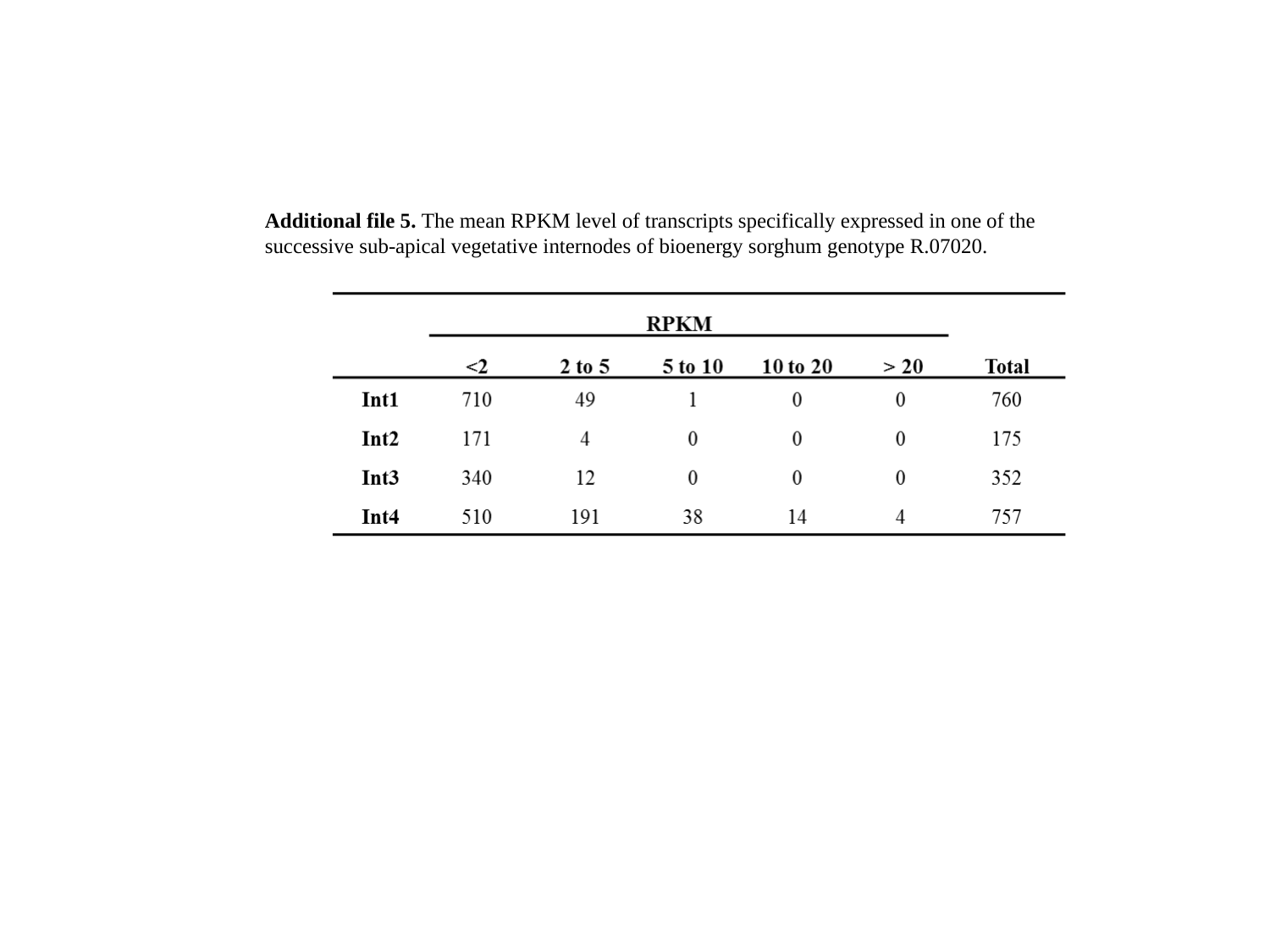

Additional file 5. The mean RPKM level of transcripts specifically expressed in one of the successive sub-apical vegetative internodes of bioenergy sorghum genotype R.07020.
