# Supplementary material for: Dynamics of gene expression during development and expansion of vegetative stem internodes of bioenergy sorghum
Source: Biotechnol Biofuels. 2017 Jun 21;10:159. doi: 10.1186/s13068-017-0848-3 (PMC5480195; doi:10.1186/s13068-017-0848-3)
Supplement: Supplementary file 6 — Additional file 6. Validation of RNA-seq results by qPCR. Expression relative to the level in Int1. [file 13068_2017_848_MOESM6_ESM.pptx]

## Slide 1
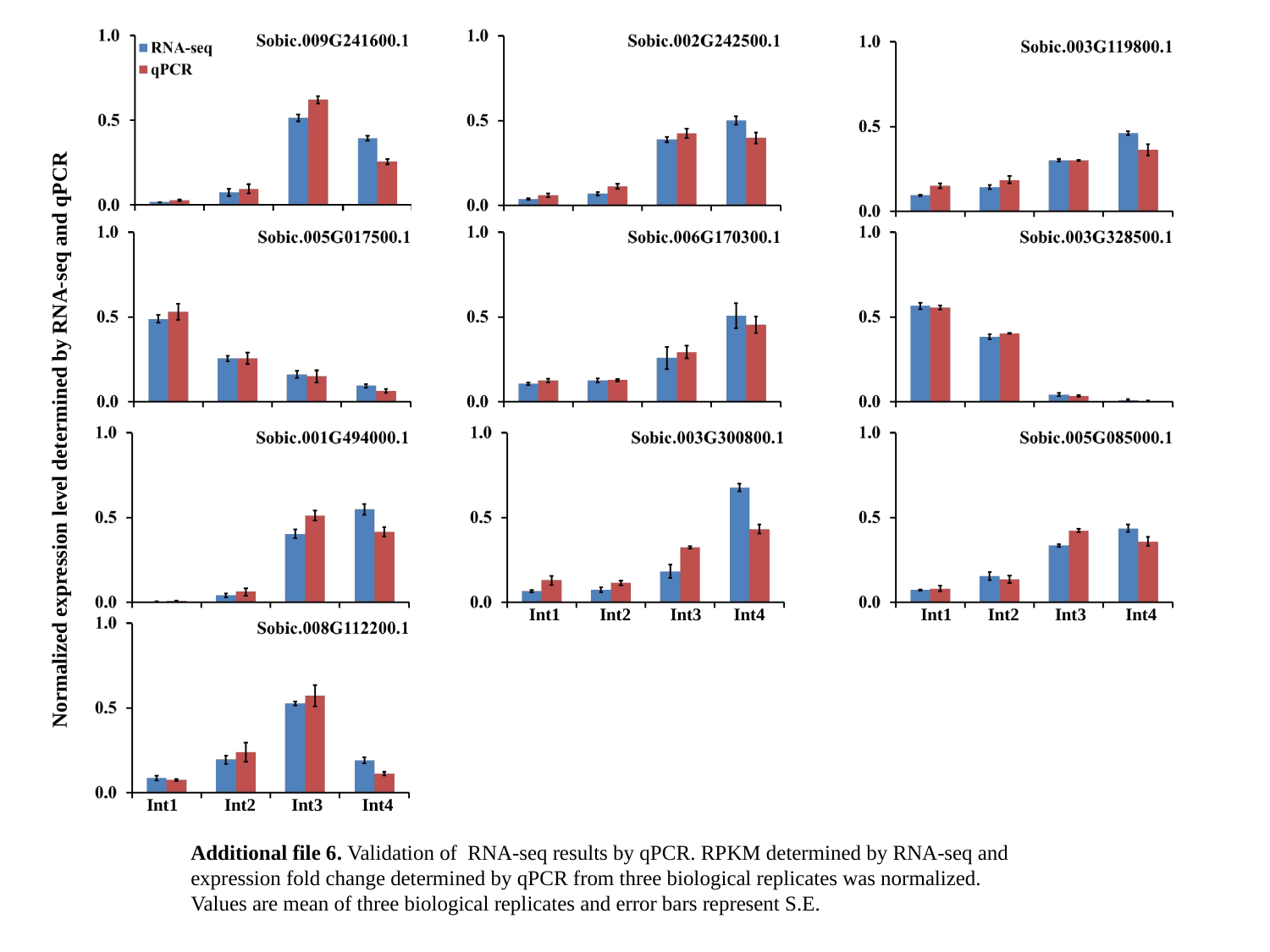

Int1
Int2
Int3
Int4
Int1
Int2
Int3
Int4
Int1
Int2
Int3
Int4
Normalized expression level determined by RNA-seq and qPCR
Additional file 6. Validation of RNA-seq results by qPCR. RPKM determined by RNA-seq and expression fold change determined by qPCR from three biological replicates was normalized. Values are mean of three biological replicates and error bars represent S.E.
