# Supplementary material for: Dynamics of gene expression during development and expansion of vegetative stem internodes of bioenergy sorghum
Source: Biotechnol Biofuels. 2017 Jun 21;10:159. doi: 10.1186/s13068-017-0848-3 (PMC5480195; doi:10.1186/s13068-017-0848-3)
Supplement: Supplementary file 7 — Additional file 7. The number of differentially expressed transcripts between any two successive sub-apical internodes. [file 13068_2017_848_MOESM7_ESM.pptx]

## Slide 1
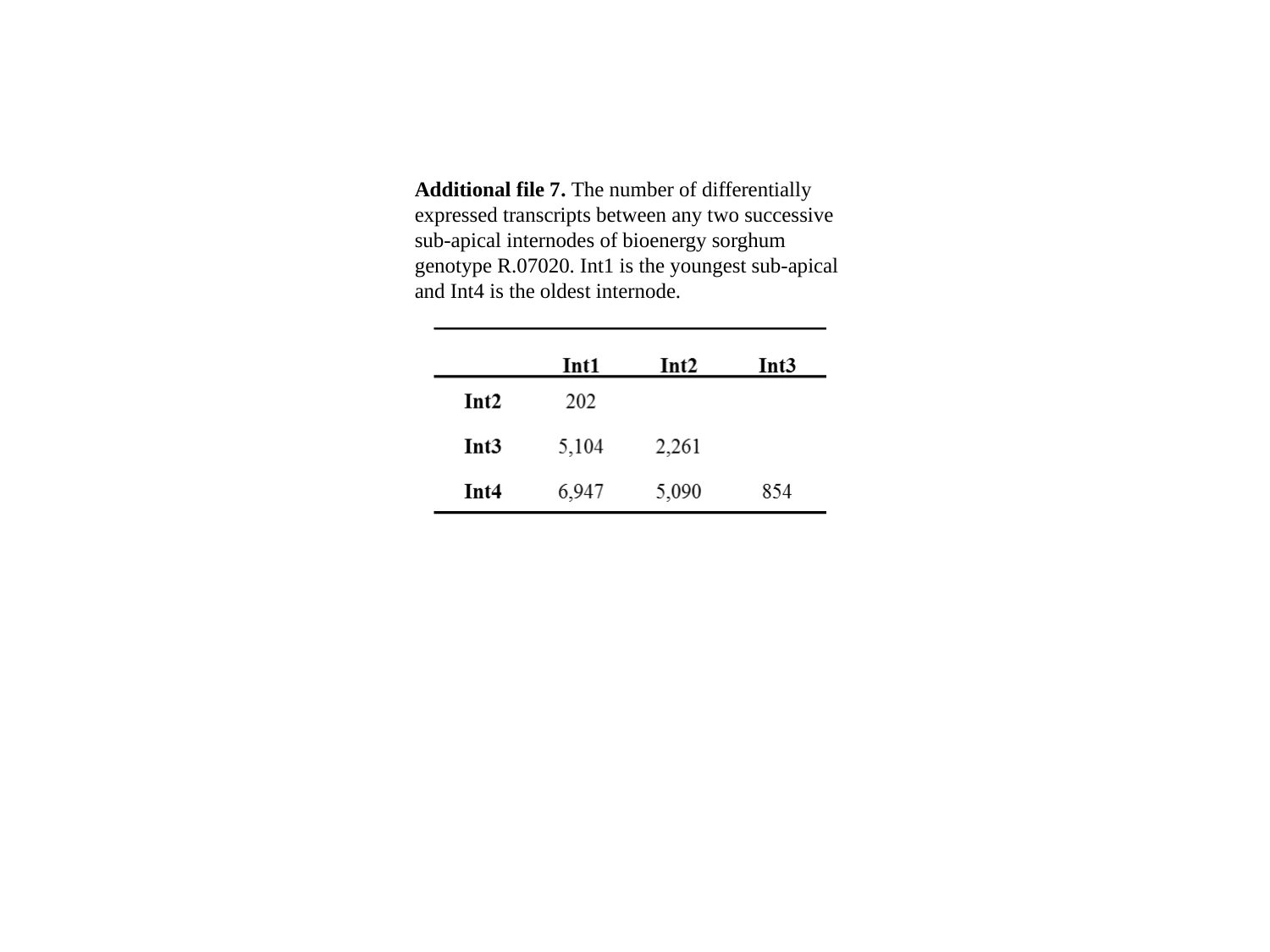

Additional file 7. The number of differentially expressed transcripts between any two successive sub-apical internodes of bioenergy sorghum genotype R.07020. Int1 is the youngest sub-apical and Int4 is the oldest internode.
