# Supplementary material for: Dynamics of gene expression during development and expansion of vegetative stem internodes of bioenergy sorghum
Source: Biotechnol Biofuels. 2017 Jun 21;10:159. doi: 10.1186/s13068-017-0848-3 (PMC5480195; doi:10.1186/s13068-017-0848-3)
Supplement: Supplementary file 9 — Additional file 9. MapMan classification of differentially expressed transcripts between four successive sub-apical internodes grouped in six clusters. [file 13068_2017_848_MOESM9_ESM.pptx]

## Slide 1
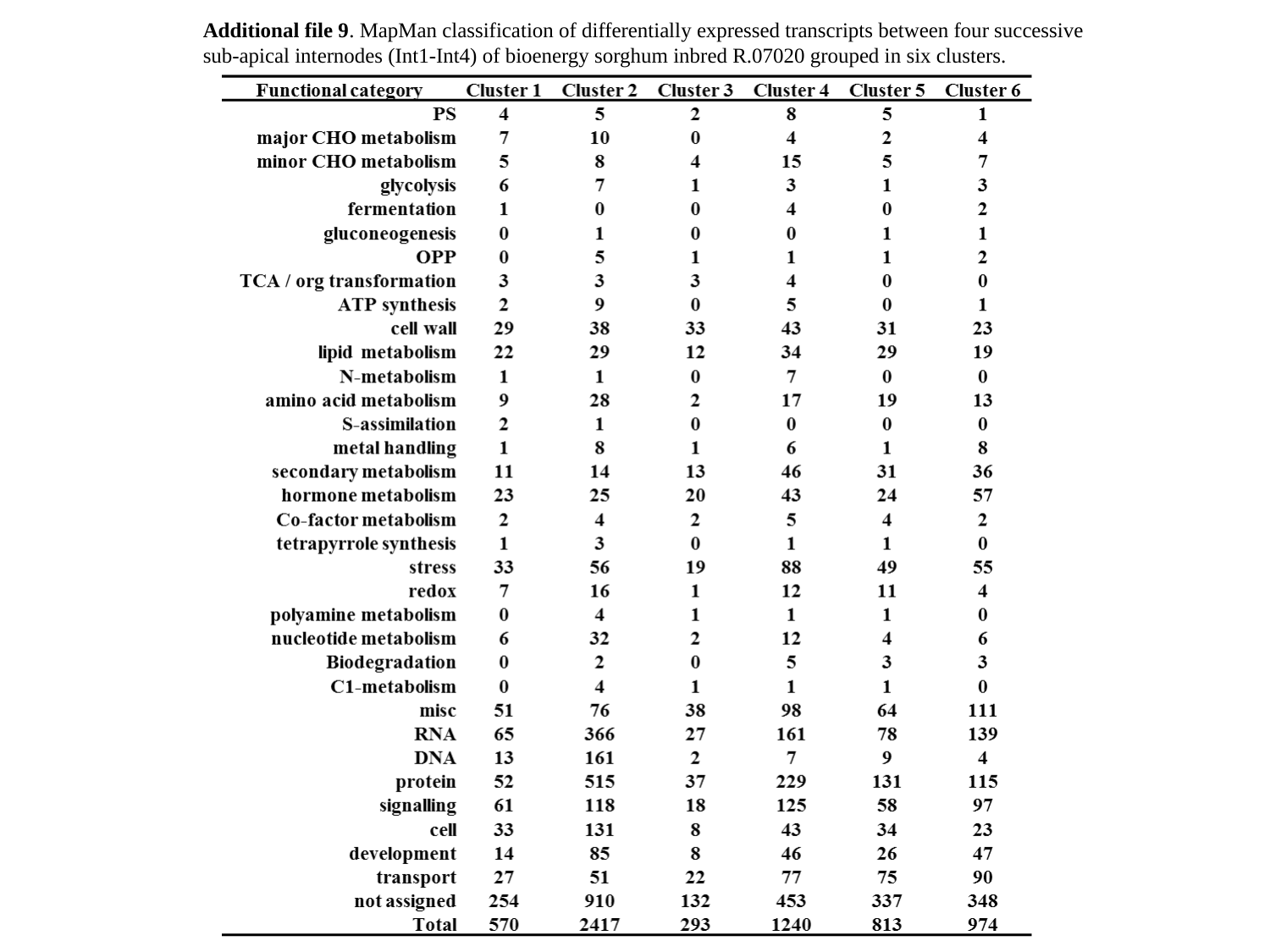

Additional file 9. MapMan classification of differentially expressed transcripts between four successive sub-apical internodes (Int1-Int4) of bioenergy sorghum inbred R.07020 grouped in six clusters.
