# Supplementary material for: Dynamics of gene expression during development and expansion of vegetative stem internodes of bioenergy sorghum
Source: Biotechnol Biofuels. 2017 Jun 21;10:159. doi: 10.1186/s13068-017-0848-3 (PMC5480195; doi:10.1186/s13068-017-0848-3)
Supplement: Supplementary file 10 — Additional file 10. MapMan functional groups with at least ten differentially expressed genes in one of the six clusters. [file 13068_2017_848_MOESM10_ESM.pptx]

## Slide 1
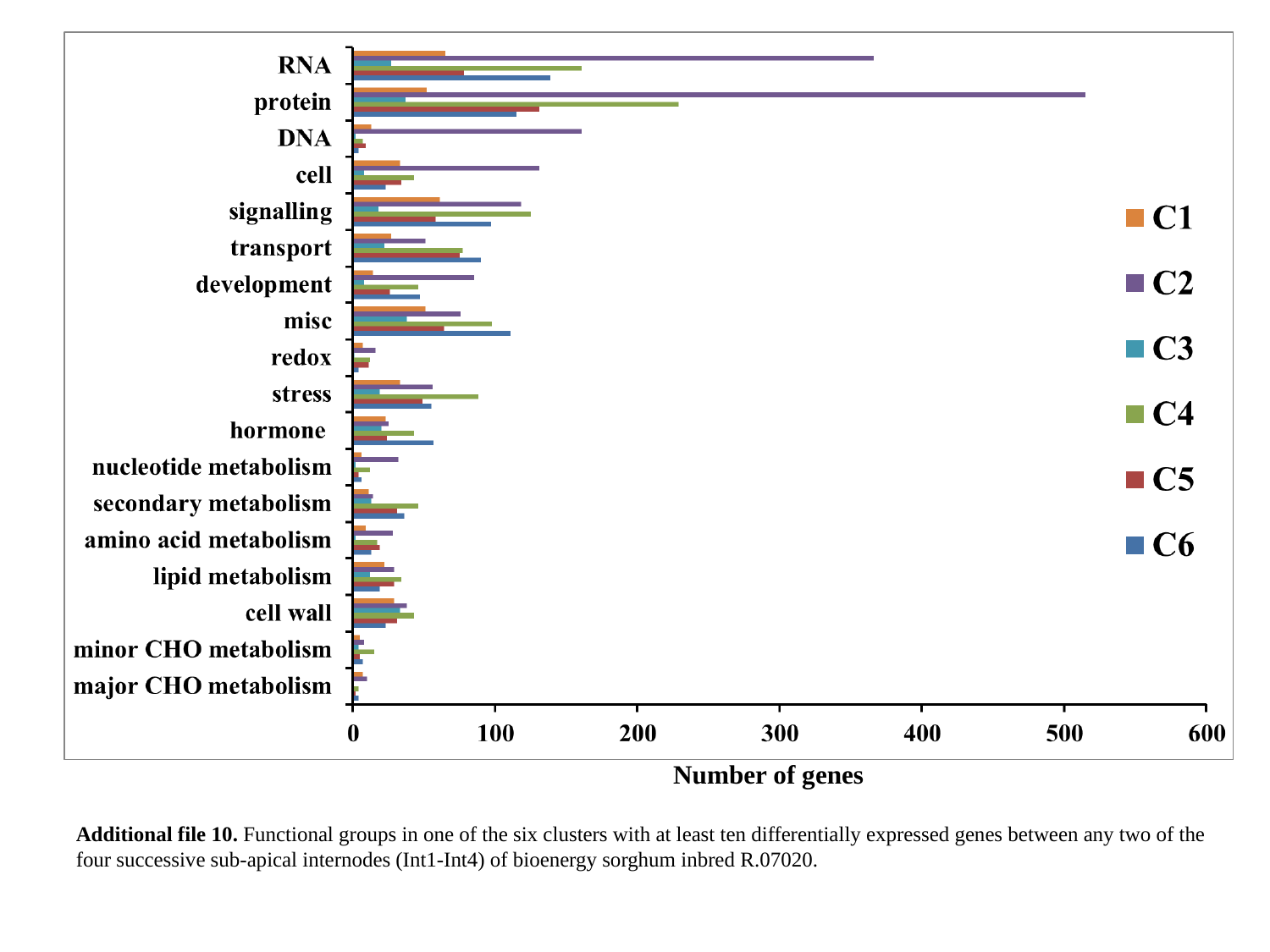

Number of genes
Additional file 10. Functional groups in one of the six clusters with at least ten differentially expressed genes between any two of the four successive sub-apical internodes (Int1-Int4) of bioenergy sorghum inbred R.07020.
