# Supplementary material for: Dynamics of gene expression during development and expansion of vegetative stem internodes of bioenergy sorghum
Source: Biotechnol Biofuels. 2017 Jun 21;10:159. doi: 10.1186/s13068-017-0848-3 (PMC5480195; doi:10.1186/s13068-017-0848-3)
Supplement: Supplementary file 11 — Additional file 11. The expression level in RPKM of trehalose biosynthesis genes in four sub-apical internodes genes between any two of the four successive sub-apical internodes. [file 13068_2017_848_MOESM11_ESM.pptx]

## Slide 1
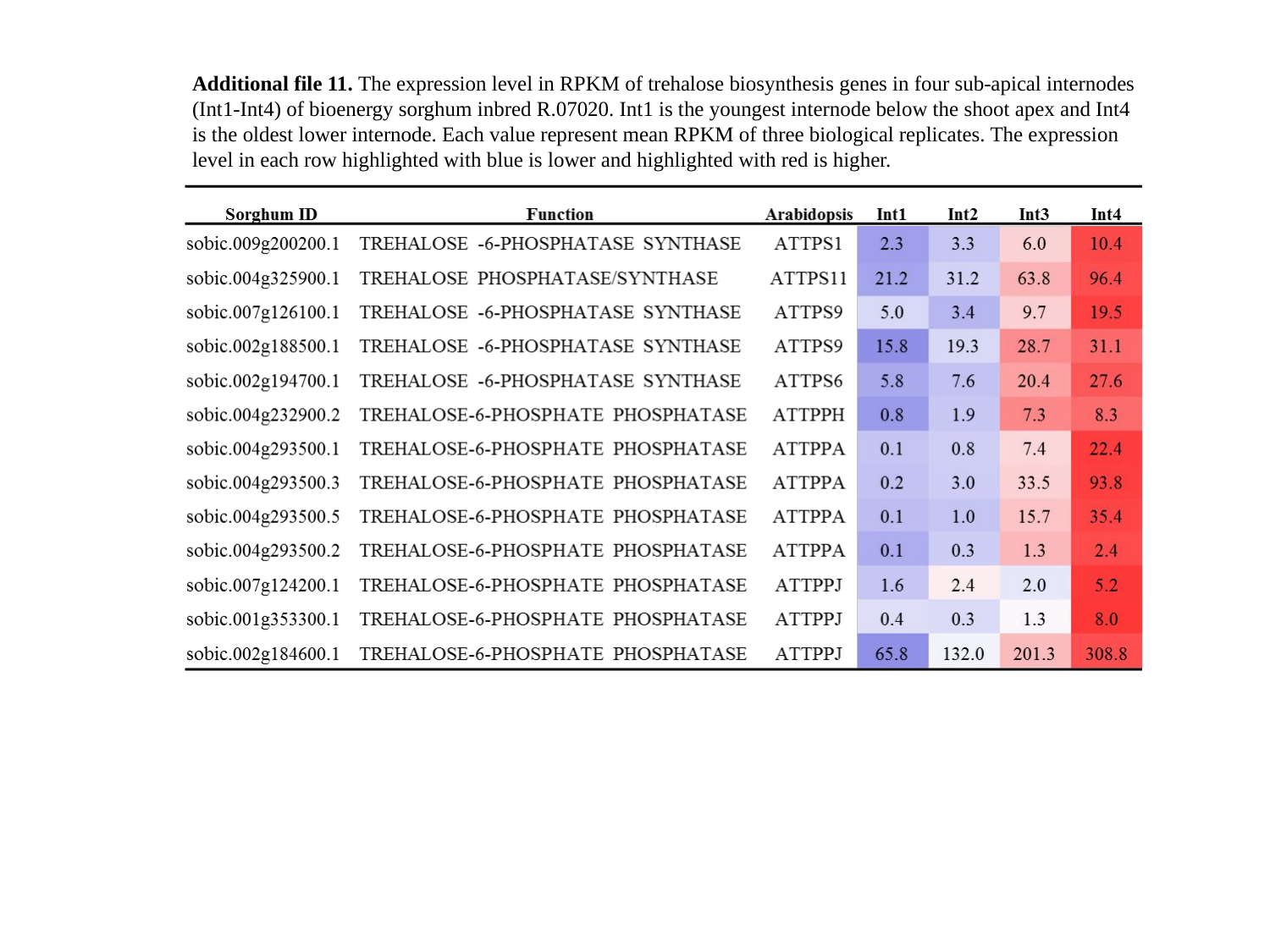

Additional file 11. The expression level in RPKM of trehalose biosynthesis genes in four sub-apical internodes (Int1-Int4) of bioenergy sorghum inbred R.07020. Int1 is the youngest internode below the shoot apex and Int4 is the oldest lower internode. Each value represent mean RPKM of three biological replicates. The expression level in each row highlighted with blue is lower and highlighted with red is higher.
