# Supplementary material for: Dynamics of gene expression during development and expansion of vegetative stem internodes of bioenergy sorghum
Source: Biotechnol Biofuels. 2017 Jun 21;10:159. doi: 10.1186/s13068-017-0848-3 (PMC5480195; doi:10.1186/s13068-017-0848-3)
Supplement: Supplementary file 12 — Additional file 12. Plant hormone metabolism and signaling genes differentially expressed between four sub-apical internodes of bioenergy sorghum inbred R.07020. [file 13068_2017_848_MOESM12_ESM.pptx]

## Slide 1
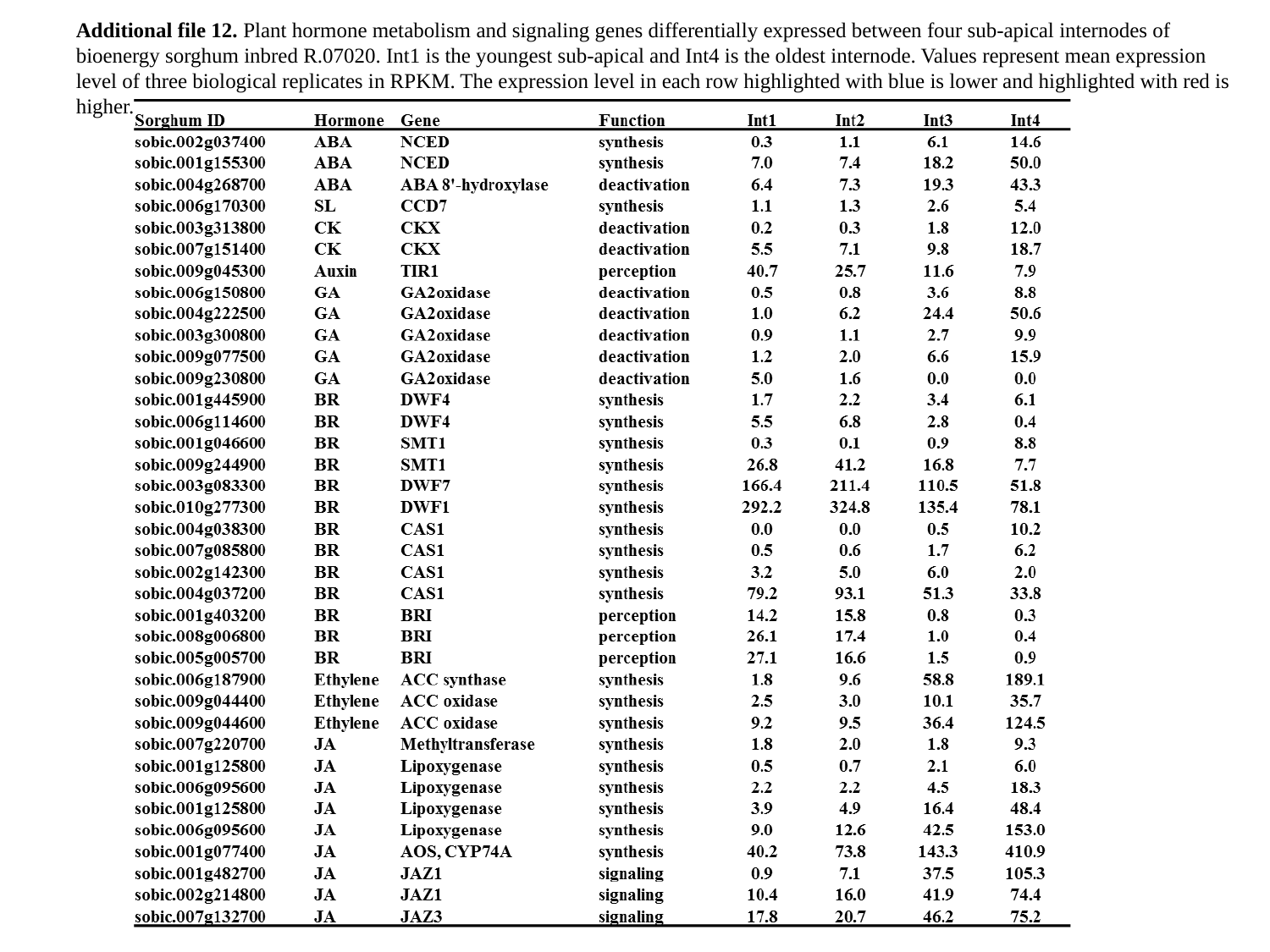

Additional file 12. Plant hormone metabolism and signaling genes differentially expressed between four sub-apical internodes of bioenergy sorghum inbred R.07020. Int1 is the youngest sub-apical and Int4 is the oldest internode. Values represent mean expression level of three biological replicates in RPKM. The expression level in each row highlighted with blue is lower and highlighted with red is higher.
